# Advanced Nano-Drug Delivery Systems in the Treatment of Ischemic Stroke

**DOI:** 10.3390/molecules29081848

**Published:** 2024-04-18

**Authors:** Jiajie Zhang, Zhong Chen, Qi Chen

**Affiliations:** 1 Key Laboratory of Neuropharmacology and Translational Medicine of Zhejiang Province, School of Pharmaceutical Sciences, Zhejiang Chinese Medical University, Hangzhou 310053, China; 202321126811230@zcmu.edu.cn (J.Z.); chenzhong@zju.edu.cn (Z.C.); 2 Interdisciplinary Institute for Medical Engineering, Fuzhou University, Fuzhou 350108, China

**Keywords:** nanoparticles, ischemic stroke, nano-drug delivery system, blood–brain barrier

## Abstract

In recent years, the frequency of strokes has been on the rise year by year and has become the second leading cause of death around the world, which is characterized by a high mortality rate, high recurrence rate, and high disability rate. Ischemic strokes account for a large percentage of strokes. A reperfusion injury in ischemic strokes is a complex cascade of oxidative stress, neuroinflammation, immune infiltration, and mitochondrial damage. Conventional treatments are ineffective, and the presence of the blood–brain barrier (BBB) leads to inefficient drug delivery utilization, so researchers are turning their attention to nano-drug delivery systems. Functionalized nano-drug delivery systems have been widely studied and applied to the study of cerebral ischemic diseases due to their favorable biocompatibility, high efficiency, strong specificity, and specific targeting ability. In this paper, we briefly describe the pathological process of reperfusion injuries in strokes and focus on the therapeutic research progress of nano-drug delivery systems in ischemic strokes, aiming to provide certain references to understand the progress of research on nano-drug delivery systems (NDDSs).

## 1. Introduction

With the improvement of living standards, people’s diets have become more refined, often high in oil, fat, and sugar. This has increased the burden on cardiovascular health, leading to a rise in conditions such as hypertension, diabetes, and atherosclerosis [1,2,3,4]. These diseases are major contributors to the increasing frequency of strokes year by year. Strokes have consequently become the second leading cause of death worldwide, attributed to its narrow therapeutic window, high mortality and disability rates, and high recurrence rate [5]. Strokes are divided into ischemic and hemorrhagic strokes, with ischemic strokes (ISs) accounting for 71% of all strokes [6]. ISs are caused by vascular embolism, which leads to ischemia and hypoxia in brain tissue. The only FDA-approved treatment for an IS is tissue plasminogen activator (tPA), which is used to treat vascular embolisms by restoring blood flow through intravenous thrombolysis. However, a reperfusion injury has greater damage to cerebral neurons, triggering a cascade of events, mainly including, energy failure, loss of cellular ionic homeostasis, excitotoxicity, mitochondrial function impairment, reactive oxygen species (ROS) generation, and immune cell infiltration, resulting in neural inflammation [7,8,9].

As the world’s most populous country, China accounts for approximately one-third of global stroke deaths, posing a significant obstacle to people’s lives and health. Consequently, research on stroke treatment has become a prominent focus in China [10]. Currently, the main clinical modalities used for stroke treatment include surgical thrombolysis and pharmacological thrombolysis [11]. The intravenous thrombolysis of tPA within 4.5 h is effective in clinical treatment, and a thrombectomy performed within 24 h also yields benefits. However, these interventions carry a narrow therapeutic window and entail a certain risk of hemorrhage. Effective recovery is achieved in only a small percentage of patients due to time constraints. The reopening of blood flow can result in a reperfusion injury, substantial ROS production, and an inflammatory response that damages neurons [12,13,14]. To date, many studies report that the inhibition of neuroinflammation is effective in alleviating secondary injury after IS reperfusion [15,16]. The infiltration of immune cells, such as microglia, astrocytes, and NK cells, is the main cause of inflammation resulting from a reperfusion injury in ISs [17,18]. Among them, microglia mainly consist of M1 and M2 types, and it has been indicated that the transition from pro-inflammatory M1 microglia to anti-inflammatory M2 type is conducive to the recovery of stroke prognosis [19]. Moreover, the suppression of neuroinflammation and scavenging of ROS are key factors in enhancing prognostic recovery from strokes. Simultaneously, the salvage of ischemic penumbra neurons can be accomplished through the use of neuroprotective agents [20]. Edaravone, a free-radical scavenger commonly used in clinical treatment, can reduce BBB damage and inhibit inflammation. However, it faces significant challenges, including difficulty in crossing the BBB, poor bioavailability, and rapid clearance from the blood, all of which contribute to low drug efficacy [21]. To address these challenges, researchers have initiated studies in nanotechnology, aiming to convert drugs into nanoforms to enhance drug utilization and reduce side effects, thereby achieving the therapeutic goal [22].

For the past few decades, nanotechnology has been developing rapidly, and nano-drug delivery systems have shown great advantages [23]. Nano-drug delivery systems can improve the solubility of insoluble drugs, enhance the stabilization, and prolong the half-life in the body. The precise targeting and control of drug release could also be realized through additional targeting fragments or surface modifications in nano-drug delivery systems, ultimately achieving the purpose of increasing efficiency and reducing toxicity [24,25,26,27,28]. The BBB after an IS is somewhat compromised, with an increase in permeability, but not enough to fail completely and still have a strong ability to insulate against external substances. Fortunately, the application of nanotechnology shows large potential and a bright future for nano-drug delivery, facilitating improved drug utilization across the BBB and into the brain [29,30]. Interestingly, carriers can also treat diseases, for example, Prussian blue (PB), which has an ROS-scavenging effect [31]. This review briefly introduces the pathologic process of reperfusion in ISs and highlights recent studies of NDDSs in treating brain ischemic reperfusion injuries, aiming to offer references that could help bridge the gap between laboratory research and clinical application.

## 2. The Pathophysiology of ISs

**Figure 1:**
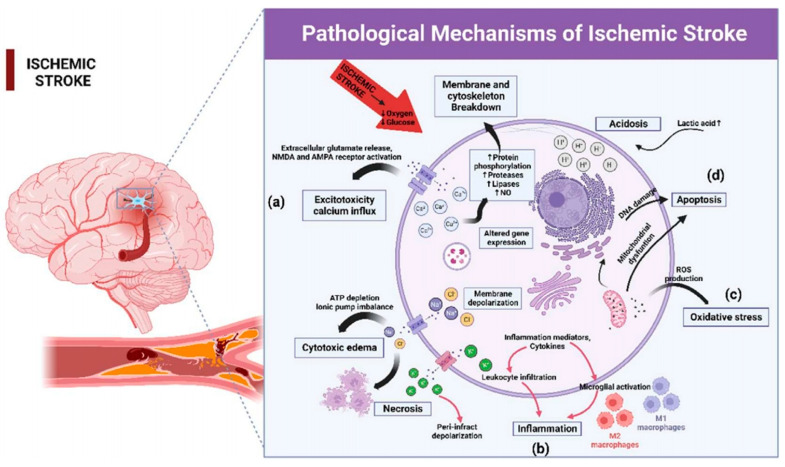
Pathologic mechanisms and cascading injury processes in ISs [35]. (**a**) Calcium overload leads to neuroexcitotoxicity; (**b**) Inflammation; (**c**) Mitochondrial dysfunction leads to oxidative stress; (**d**) Apoptosis; Copyright 2023, Elsevier.

Multiple complex pathological mechanisms are involved in brain damage caused by ISs [32,33,34]. Understanding these complex mechanisms not only deepens our understanding of the disorders but also aids researchers in facilitating the translation from the laboratory to clinical practice. At the onset of an IS, local ischemia and brain hypoxia lead to metabolic disorders in the intracerebral microenvironment, which then trigger a series of cascading injury processes, as shown in Figure 1 [35]. IS pathology begins with cerebral ischemia and hypoxia, culminating in neuronal apoptosis [36]. Energy failure is due to ischemia and hypoxia, which stimulate neuronal depolarization to release large amounts of glutamate. This process causes calcium influx and results in mitochondrial dysfunction [37]. At the same time, the accumulation of glutamate induces cellular neuro-excitotoxicity, the massive release of reactive oxygen and nitrogen species (RONS), and the penetration of peripheral immune cells to the brain [17,38,39]. After vascular reperfusion, oxygen and energy supply are restored, but mitochondrial dysfunction and the inability to process excess oxygen disrupt the balance between RONS production and clearance [40,41]. Excessive RONS would induce oxidative stress, which damages endothelial cells, and causes platelet aggregation and adhesion in microvessels, leading to the reformation of the thrombus and ultimately resulting in cerebral edema and bleeding [42,43,44]. This in turn further promotes immune cell infiltration, exacerbating the inflammatory response and causing neuroinflammation [45]. These reactions interact with each other and do not exist singularly. When an IS occurs, thrombolysis within a few hours can alleviate brain damage; however, secondary damage from cerebral ischemia–reperfusion poses a greater challenge [46,47].

Although cerebral neuronal damage in the core area of ischemia is irreversible, the penumbral area of cerebral ischemia affected by the spread of ROS and immune cell infiltration is recoverable [48,49]. Therefore, the semi-dark band in cerebral ischemic is an important target area for improving prognosis and restoring neurological function [5,50,51]. The cerebral ischemic penumbra can be rescued by suppressing the immune–inflammatory response and attenuating oxidative stress [52,53,54]. Some studies have found that administering exogenous mitochondria to the ischemic area can alleviate mitochondrial dysfunction. Exogenous mitochondria can use endogenous mechanisms to repair cellular damage, potentially resulting in therapeutic effects on the nerves after an IS [55,56,57]. 

In this section, we mainly focus on excitotoxicity, oxidative stress, and neural inflammation. Other mechanisms of injury will not be discussed further.

### 2.1. Excitotoxicity

Excitatory amino acids, such as glutamate and aspartate, serving as central neurotransmitters, play critical roles in transmitting messages in the nervous system [58,59]. Among them, glutamate is widely distributed throughout the brain and exerts a significant excitatory effect on central neurons [37]. Under physiological conditions, the brain microenvironment maintains homeostasis without excessive glutamate production, which can be metabolically regulated. However, when an ischemic stroke occurs, because the blood supply is interrupted, neurons are deprived of energy and oxygen, resulting in decreased ATP production. This leads to the abnormal functioning of the NA^+^-K^+^ ion pump in the cell membrane [60]. Meanwhile, N-methyl-D-aspartate receptors (NMDAR) regulate ion-gated pathways by binding glutamate, thereby exerting excitatory effects on neuronal cells [42,61]. Unfortunately, when substantial amounts of glutamate binding to aspartate receptors, it triggers a significant inward flow of Ca^2+^, resulting in calcium overload and NO^−^ production [62]. NO^−^ reacts with O^2−^ to produce the destructive OONO^−^, exacerbating BBB damage and brain injury [63,64,65]. In conclusion, excitotoxicity arises from uncontrolled glutamate release and calcium overload, triggering a series of effects that ultimately result in the death of neurons [66,67]. Thus, excitotoxicity represents an important molecular mechanism contributing to IS damage [68].

### 2.2. Oxidative Stress

As a downstream consequence of an excitatory injury, oxidative stress emerges as another major molecular mechanism contributing to ISs [23]. Oxidative stress occurs when the oxidative homeostasis system is disrupted due to the massive release of free radicals [69]. Excessive free radicals cause cellular damage, DNA damage, skeleton disruption, and lipid peroxidation, ultimately resulting in neuronal damage and brain death [70,71]. In particular, the free radical blowout after reperfusion exposes neurons to even more severe challenges [72,73]. In addition, free radicals can stimulate the secretion of cytokines as well as the expression of adhesion molecules, which mediate inflammatory and immune responses, thereby exacerbating a brain tissue reperfusion injury [74].

### 2.3. Neural Inflammation

The inflammatory response plays a pivotal role in the pathological events associated with brain damage and repair processes in ischemic strokes [75]. Various inflammatory cells participate in the inflammatory reaction. Microglia, astrocytes, neutrophils, NK cells, and other immune cells constitute the major effector cells during an IS [76,77]. Here, we take microglia and NK cells as examples. Microglia are intrinsic immune cells residing in the brain that play an important role in CNS diseases and that can be classified into M1 and M2 phenotypes [78,79]. M1 microglia secrete massive ROS, pro-inflammatory factors, and protein hydrolases to promote inflammation and exacerbate neuronal injuries, whereas M2 microglia secrete anti-inflammatory factors, which attenuate inflammatory injuries and improve the recovery of brain injuries [75,80]. While the activation of microglia is initially intended to protect neuronal cells, their overactivation can lead to harmful inflammation and neuronal death [81]. Numerous studies have shown that facilitating the conversion of microglia from M1 to M2 types can reduce inflammation and improve stroke prognosis [52,82,83,84,85]. For NK cells, they exacerbate stroke injuries by increasing local inflammation and neuronal hyperactivation, ultimately resulting in neuronal cell death [86,87]. The reason for this is that NK cells, which are common immune cells in the body, are actively recruited into the site of injury when an IS occurs. Simultaneously, NK cells secrete a large number of cytokines that promote inflammatory damage, ultimately leading to irreversible brain damage. Although immune cells that initially infiltrate into brain tissue exacerbate brain damage and neurological dysfunction, they also later play beneficial roles, such as promoting glial scarring and the phagocytosis of debris, which are essential for wound healing [88].

## 3. Advanced NDDSs for ISs

Prolonging the time window of treatment and mitigating a secondary reperfusion injury are ideal for addressing ISs [89]. Fortunately, recent advances in NDDSs have emerged, offering promising possibilities for enhancing IS reperfusion therapy [90]. NDDSs are known for their small size, large surface area, controlled release, targeted modification, and high ability to penetrate the BBB [91,92]. In addition, NDDSs can be utilized to load hydrophobic, hydrophilic, and gene-based drugs while also functioning as labeling probes for tracer imaging of ISs [93,94]. In the realm of advanced NDDSs for an ischemic stroke (IS), common types include polymers, inorganic nanoparticles, liposomes, and cell membrane-coated nanoparticles [95,96].

### 3.1. Polymers

**Figure 2:**
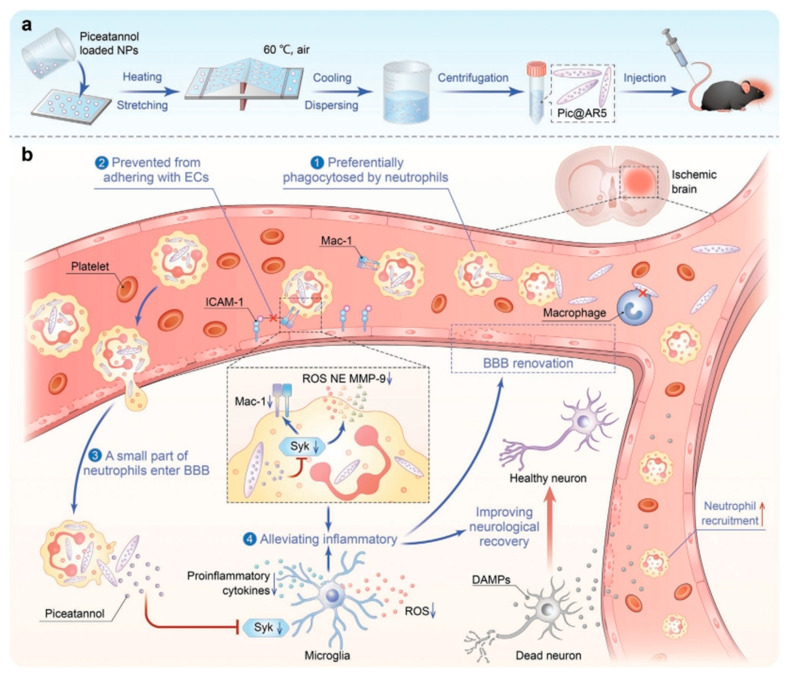
(**a**) Preparation of rod-like nanoparticles by solvent evaporation and film stretching methods; (**b**) Rod-like nanoparticles (Pic@AR5) were specifically phagocytosed by neutrophils, followed by the release of pisaclitaxel which blocked the Syk pathway and reduced the expression of β2 integrins, thus preventing the adhesion of most neutrophils to endothelial cells. Meanwhile, a small number of Pic@AR5-carried neutrophils enter the BBB and release piceatannol in the ischemic zone, inhibiting the Syk pathway in microglia and attenuating microglia-mediated neuroinflammation [106]. Copyright 2023, Wiley-VCH.

Polymers offer broad prospects for addressing drug delivery for ISs [29]. They excel as NDDSs for treating CNS disorders due to their exceptional qualities, including excellent biodegradability, high biocompatibility, and minimal toxicity [97,98,99]. There are numerous polymers, among which PLGA, poly(ethylene glycol) (PEG), and poly(lactic acid) (PLA) are common and the most widely studied [100]. The appropriate modification of polymeric nanoparticles can enhance brain targeting and achieve drug enrichment in the target region [100]. PLGA is synthesized from the polymerization of two monomers: lactic acid and hydroxyacetic acid. It is an FDA-approved drug excipient known for its high encapsulation efficiency and excellent biocompatibility [101,102]. Previous studies by Wang et al. have demonstrated that epidermal growth factor (EGF) and erythropoietin (EPO) have a stimulatory effect on stem cells, further promoting tissue repair [103,104]. Based on this premise, Wang et al. proceeded to explore the encapsulation of pegylated EGF in PLGA nanoparticles as well as the formulation of hydrogels through the encapsulation of EPO within two-phase particles composed of PLGA and poly(sebacic acid). This system, through the epidermis to the ischemic lesion area, was observed to effectively traverse the BBB and stimulate the differentiation of endogenous neural stem cells. This ultimately facilitated the notable restoration of tissue and nerve function in the mice [105]. A prospective approach to treating ISs could involve preventing neutrophil infiltration. Song et al. developed a rod-shaped PLGA nanoparticle loaded with piceatannol for targeted intervention in neutrophil–endothelial cell interactions. They drew inspiration from the fact that neutrophils exhibit a preference for phagocytosing elongated particles and that rod-shaped PLGA is readily phagocytosed by neutrophils [106] (Figure 2a). This strategy can protect neurons and attenuate stroke damage to the brain by reducing neutrophil adhesion to endothelial cells and reducing immune infiltration (Figure 2b). In addition, PLGA can be used to form nanoparticles containing superparamagnetic iron oxide and Cy7.5, serving a pivotal role in MRI and fluorescence imaging [107].

Surface modification with PEG prolongs in vivo circulation and decreases the immunogenicity of nanoparticles [108]. Curcumin is known as an anti-inflammatory and antioxidant, but weak hydrophilicity and chemical instability make its application challenging [109]. Wang et al. used an amphiphilic copolymer consisting of PEG and PLA to prepare NDDS-encapsulating curcumin, which improves the stability of curcumin in blood circulation. The NDDS could protect the BBB by inhibiting the decrease in tight junction proteins [110]. The results showed that this NDDS inhibited M1 microglia polarization and inflammatory damage, thereby further promoting the functional recovery of brain tissue in mice. In another interesting study, PEG was used for modification to prepare pH-sensitive polymers loaded with rapamycin (RAPA), named RAPA@NPs. A pH-sensitive link was employed to achieve acid-triggered drug release, Ce6 was selected as the near-infrared imaging agent, and Gd^3+^ chelator was chosen for implementing bimodal imaging [111]. The results showed that the prepared RAPA@NPs not only overcame the drawbacks, such as the poor solubility of RAPA itself, thereby improving the therapeutic efficiency of RAPA, but also exhibited good biocompatibility and acid-enhanced bimodal imaging capabilities. RAPA@NPs preferentially aggregated at the ischemic site of the brain and achieved significant neuroprotective effects. The study provides a promising NDDS for drug tracking, treatment, and the early diagnosis of an IS as well as a reference towards the accurate imaging and treatment of other diseases [111]. Similarly, Ding et al. developed PEG coupled with urokinase (UK), wherein UK: PEG-UK was mixed in a 1:1 ratio [112]. The results demonstrated that this NDDS provided dual targeting of the macro-vasculature and the microcirculation, showing excellent neurologic function scores and smaller infarcts in the MCAO model area.

Dendritic macromolecules are excellent candidates for biological and pharmaceutical applications [113,114,115]. Among them, polyamidoamine dendrimer (PAMAM) is the most widely studied dendrimer [116]. This can be attributed to their controllable size structure, good water solubility, extensive internal drug-carrying space, and the potential for external modification [117,118]. However, PAMAM suffers from susceptibility to clearance by the reticuloendothelial system (RES) and a lack of targeting ability [119]. Despite these limitations, PAMAM can acquire enhanced capabilities through modifying and transforming into more desirable carriers. Examples include the conjugation of ligands such as PEG, folate analogs, protein analogs (transferrin, lactoferrin), amino acids, and peptides [120,121,122,123]. Successful NDDSs based on PAMAM for the treatment of neuroinflammation and ISs have been reported, yielding satisfactory therapeutic outcomes [124,125,126,127].

Collectively, the excellent properties of polymers allow them to be used as an NDDS, taking advantage of the microenvironmental characteristics for responsive release [111,128,129,130].

### 3.2. Inorganic Nanoparticles

Inorganic nanoparticles possess unique physicochemical properties [90]. They are characterized by a controllable structure, modifiable surface, and high loading efficiency, showing great research value in disease treatment and diagnosis [131,132,133]. For instance, gold nanoparticles are easy to synthesize and functionalize for versatile applications, possessing specific physical, electrical, magnetic, and optical properties [134,135]. Silica and iron have natural properties that allow them to work for MRI tracking [136,137]. Fe_3_O_4_ and CeO_2_ have enzyme-like activities that contribute positively to the removal of excess ROS [138,139,140].

Expanding on last paragraph, given the capability of gold nanoclusters to penetrate the BBB and the anti-inflammatory and antioxidant properties of dihydrolipoic acid, Xiao et al. synthesized functionalized gold nanoclusters carrying dihydrolipoic acid [141,142,143,144]. After the gold nanoclusters carried the drug through the BBB and into the brain, the dihydrolipoic acid underwent reduction and exerted its scavenging effects [144]. By regulating microglia and polarizing them to the M2 type, this NDDS can reduce the inflammatory response and improve the neuronal survival rate. In addition, the clinical application of gold nanoparticles for the photothermal therapy of prostate cancer has been carried out [145,146].

Magnetic iron oxide nanoparticles have garnered increasing attention due to their unique properties [94]. Liu et al. developed co-doped Fe_3_O_4_ nanoenzymes to ameliorate the RONS overload injury caused by an IS [147]. In vitro cellular experiments demonstrated that Fe_3_O_4_ nanoenzymes were effective in ameliorating neuroinflammation caused by ISs, as well as significantly reducing the infarct volume in both transient and permanent stroke models. Wang et al. developed an MRI visualization technique to track and visualize transplanted stem cells, offering valuable insights to enhance the effectiveness of IS treatment [148]. Similarly, some studies used superparamagnetic iron oxide nanoparticles (SPIO) to label stem cells for imaging tracking in IS therapy [107,149,150]. In addition, iron oxide nanoparticles can be loaded with dexamethasone and L-carnosine peptide for targeted delivery in ISs, which already yield satisfactory experimental results. It demonstrated the feasibility that iron oxide nanoparticles can be used as an NDDS for the treatment of ISs [151]. Clinical approval of iron oxide-based MRI contrast agents has been obtained in Europe and the United States; however, challenges persist in their clinical translation [152]. Overall, iron oxide-based nanoparticles hold excellent potential for application and clinical translation [153,154,155,156,157].

**Figure 3:**
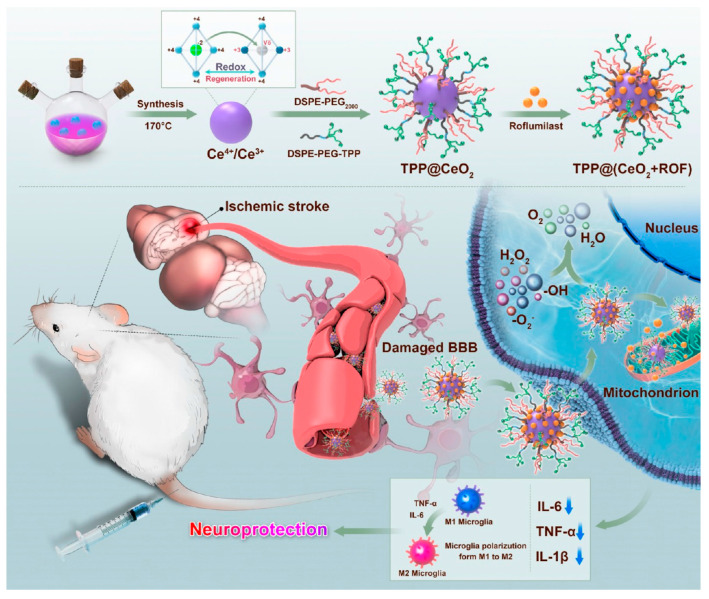
Schematic illustration of mitochondria-targeted cerium nanoenzymes for the therapy of ISs. Neuroprotective effects are achieved by attenuating oxidative stress and modulating microglia phenotype [162]. Copyright 2024, American Chemical Society.

CeO_2_ nanoparticles are effective free radical scavengers [158,159]. Li et al. developed CeO_2_ nanoparticles loaded with butylphthalide (NBP-CeO_2_ NPs), which have neuroprotective effects for ISs [160]. The results of long-term experiments demonstrated that NBP-CeO_2_ NPs can promote vascular repair and improve the behavioral functions of mice. The combination of free radical scavenging and neurovascular repair can significantly reduce reperfusion injury, and this treatment approach holds promise for applications in ISs. He et al. developed CeO_2_ nanoparticles encapsulated with zeolitic imidazolate framework-8-capped (CeO_2_@ZIF-8). Compared to bare CeO_2_, the CeO_2_@ZIF-8 exhibited higher stability and longer blood circulation time under physiological conditions [161]. Moreover, due to its peroxidase-like properties, ZIF-8 enhances the free radical scavenging ability of CeO_2_@ZIF-8 compared to free CeO_2_, showing excellent preventive and therapeutic effects in neuroprotective therapy for ISs. Liao et al. introduced the concept of mitochondrial microenvironmental regulation and developed an NDDS based on CeO_2_-targeting mitochondria [162] (Figure 3). Mitigating oxidative stress injury can be achieved by regulating the mitochondrial microenvironment to promote ischemic recovery. 

In addition, manganese-based nanoparticles, including MnO_2_ and Mn_3_O_4_, have also received much attention in ISs [52,163,164]. In the news from 2023, during the European Congress of Radiology (ECR) in Vienna, Austria, the GE Healthcare Group announced that the completion of Phase I subject recruitment for its pioneering manganese-based macrocyclic magnetic resonance imaging (MRI) contrast agent in early clinical development program. To sum up, the integration of inorganic nanoparticles into ischemic treatment approaches holds great potential for enhancing therapeutic outcomes through the modulation of cellular processes and microenvironmental factors.

### 3.3. Liposomes

**Figure 4:**
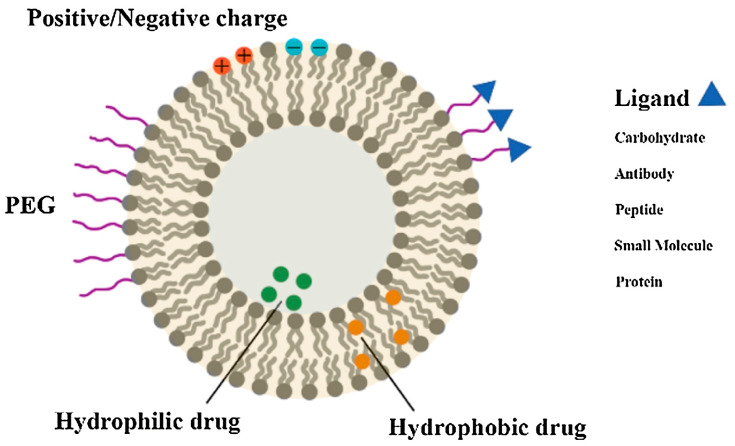
Schematic illustration of the functionalization of liposomes [166]. Copyright 2019, Elsevier.

Liposomes, discovered by Bangham in 1965, are enclosed vesicles composed of ordered lipid bilayers [165]. The bilayers consist mainly of amphiphilic phospholipids with aqueous spaces inside. Thus, components with hydrophilic properties can be encapsulated in the aqueous cavity of the liposome, while components with hydrophobic properties can be encapsulated in the lipid bilayer [166] (Figure 4). Compared to other NDDSs, studies on liposomes are relatively well established. Due to their excellent biocompatibility, high safety profile, and noteworthy therapeutic efficacy, numerous liposome formulations have received FDA-approval, signifying the progression from laboratory investigation to clinical application [167,168]. Undoubtedly, the successful translation of liposomes from the laboratory to the clinic has garnered considerable attention from researchers across diverse fields, fostering further in-depth investigations into liposomes [169,170]. 

Plain liposomes are easily cleared by the reticuloendothelial system (RES), while PEGylated liposomes extend the drug’s circulation time in the bloodstream and mitigate the risk of recognition and clearance by immune cells [171]. Thomas et al. prepared a PEGylated liposome loaded with atorvastatin, which could accumulated at the ischemic lesion area effectively, reducing infarct volume and promoting neurological recovery [172]. The use of PEGylated liposomes for delivering neuroprotective agents also improves drug bioavailability in vivo. Notable examples include citicoline [173], lycopene [174], FK506 [175], and plasminogen activators [176]. In addition, the addition of some FC fragments, transferrin, and other kinds of ligands or stimuli-responsive fragments to liposomes can achieve active targeting and responsive release [177,178,179]. To address the phenomenon of neutrophil infiltration into the ischemic zone during an IS, resulting in the release of neutrophil extracellular traps (NETs) and subsequent neuronal damage, Sun et al. developed a smart liposome with ischemic lesion targeting and ROS-responsive release [180]. The results showed that the smart liposome could regulate NETs and promote microglia transformation to the M2 type to treat ISs, resulting in a cerebral infarction area approximately one-fourth that of the saline group. Li et al. investigated methods to enhance the delivery efficiency of the commercially available drug ginkgolide B (GB) by developing a liposomal formulation that binds GB to high-lipophilic docosahexaenoic acid (DHA) (Lipo@GB-DHA) [181]. The results indicated that when compared with the free GB group, the amount of GB in the target ischemic hemisphere in the Lipo@GB-DHA group was 2.2 times higher than that in the free GB group. Additionally, the Lipo@GB-DHA group showed a smaller infarct area, better inhibition of neuronal apoptosis, and improved recovery of neurological function. Yao et al. developed a pH-responsive fluorescent liposome probe and established a correlation between fluorescent imaging and neurologic deficiency scores. The approach provided a novel approach for assessing the extent of ISs in different acidic microenvironments [182]. Noteworthly, the transnasal administration of liposomes have achieved good therapeutic results in rat IS models [183,184].

In conclusion, substantial advancements have been achieved in utilizing liposomes as an NDDS. However, problems such as instability, leakage or poor targeting, and the sudden release of drugs remain to be solved in liposomes [185]. These issues highlight the critical need for the development of advanced technologies aimed at enhancing the properties of liposomes.

### 3.4. Cell Membrane-Coated Nanoparticles

**Figure 5:**
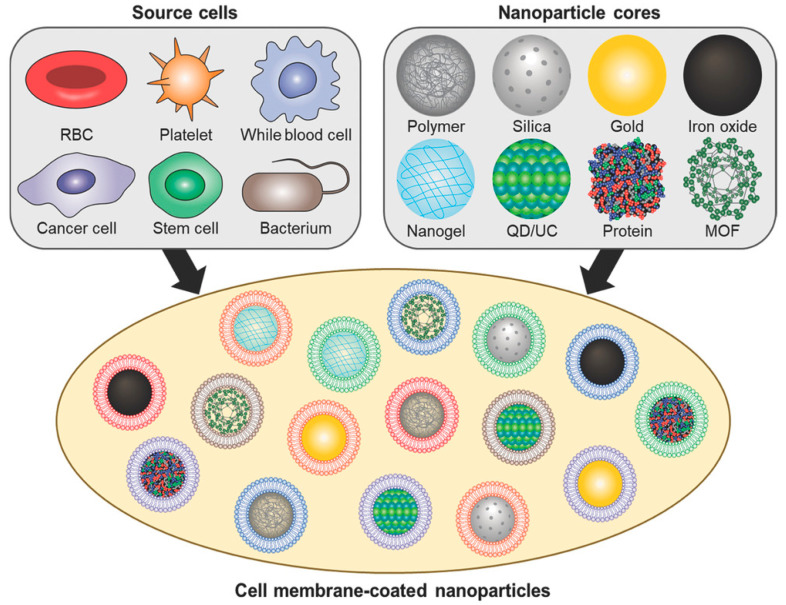
Diverse cell membrane-coated nanoparticles [188]. Copyright 2018, WILEY-VCH.

Targeted nanomedicines hold promising potential in stroke therapy. However, some of them are intrinsically foreign and face the risk of being removed by the RES [186]. In order to address this obstacle, the researchers proposed a cell membrane-coated strategy. Cell membrane-coated nanoparticles possess the unique advantage of imparting biological characteristics by hiding traditional nanoparticles in natural cell membranes [187]. By selecting different kinds of cell membranes to modify the outer layers of NDDSs, NDDSs with enhanced surface functionality can be created to achieve diverse goals [188] (Figure 5). Cell membranes from different sources have different functional characteristics, as shown in Table 1. However, cell membranes still have some limitations [189]. Fortunately, it is possible to modify some targeting proteins or peptides on the membrane surface to further improve the targeting performance and ultimately achieve better therapeutic effects [190,191].

#### 3.4.1. RBC Membrane-Coated Nanoparticles (RBC-NPs)

It is well known that RBCs are abundantly present within the blood and lack nucleus and mitochondria [189]. Consequently, the RBC membrane is easily extracted. The lifespan of RBCs in the body is about four months. During this time, the surface expression of the CD47 protein, which serves as a “do-not-eat-me” signal, prevents RBCs from being removed by the RES [203]. These characteristics of RBCs offer potential prerequisites for the development of RBC-NPs [204].

RBC-NPs were first reported by Zhang et al. in 2011 [205]. Compared with NPs coated with hydrophilic polymer PEG, which showed long circulation in vivo, the RBC-NPs retained RBCs’ biological properties, showing a longer circulation time. After that, researchers proposed diverse approaches to modify targeting peptides or ligands on the RBC membrane, which could not disrupt the RBC membrane but improve the brain targeting ability [206,207,208].

**Figure 6:**
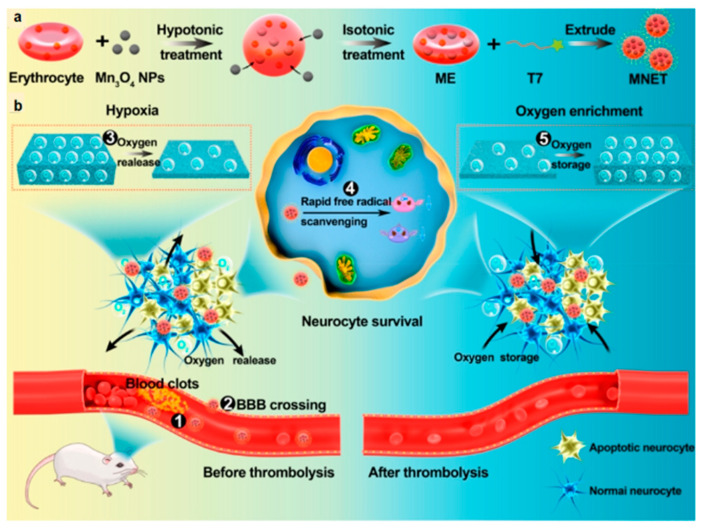
(**a**) Flowchart of MNET preparation; (**b**) Schematic illustration of oxygen sponge function and free radical scavenging of MNET in vivo. MNET crosses the BBB and accumulates at the site of infarction, with effects of releasing oxygen to relieve hypoxia and storing oxygen in a pro-oxidant environment after thrombolysis [209]. Copyright 2020, American Chemical Society.

Shi et al. developed an engineered RBC-NP named Mn_3_O_4_@nanoerythrocyte-T7 (MNET) with smart oxygen regulation and free radical scavenging [209] (Figure 6a). Hemoglobin (HB) in RBCs underwent oxygen uptake and release, functioning like an oxygen sponge. Meanwhile, Mn_3_O_4_ NPs showed high biocompatibility and multiple antioxidant enzyme activities. By combining the advantages of RBCs, Mn_3_O_4_, and T7 peptides, the rescue of the ischemic microenvironment was finally realized using MNET. More excitingly, MNET could be used for continuous treatment via the oxygen spongy effect of HB. During an IS, the oxygen spongy function of HB can provide oxygen to the hypoxic area and reduce ischemic injury (Figure 6b). Oxygen reperfusion after thrombolysis causes the production of free radicals, and HB could play an important role in absorbing excess oxygen and reducing oxidative stress. Liu et al. also developed an integrated approach for acute ISs inspired by the oxygen spongy properties of HB [210]. They corrected abnormalities in glucose metabolism and provided energy to neurons by releasing methoxatin, which acts to activate the cellular Akt/GSK-3β pathway. It can be observed that the oxygen balance of the microenvironment and glucose metabolism are important for neuronal recovery. Lv et al. designed an erythrocyte membrane delivery system (SHp-RBC-NP/NR2B9C) with cerebral ischemic region-targeting and ROS-responsive release capabilities [211]. Based on the fact that ROS are released in large quantities from the ischemic region during the pathogenesis of an IS, phenylboronic acid with ROS-responsive release capabilities was added to the SHp-RBC-NP/NR2B9C. It was verified in the rat MCAO model that the SHp-RBC-NP/NR2B9C could successfully reach the ischemic lesion, release the neuroprotectant NR2B9C in the ischemic ROS microenvironment responsively, and reduce the volume of cerebral infarcts.

In conclusion, RBC-NPs, as the earliest membrane-coated NDDS studied, have tremendous possibilities through modification [212]. They have aroused researchers’ interest, and more researchers have begun to exploit the feasibility of fabricating hybrid RBC-NPs.

#### 3.4.2. Platelet Membrane-Coated Nanoparticles

Platelets have a shorter lifespan compared to erythrocytes and are present in the blood in smaller numbers than erythrocytes [213]. Platelets, in addition to CD47, exhibit other expression clusters, such as CD55 and CD59, on their membrane surface, preventing phagocytosis and clearance by immune cells, consequently enhancing immune evasion [214]. In addition, platelets are known for their role in recognizing and repairing vascular damage and responding to inflammation. Consequently, nanoparticles encapsulated in platelet membranes can be directed toward the site of the injury for targeted delivery [215].

**Figure 7:**
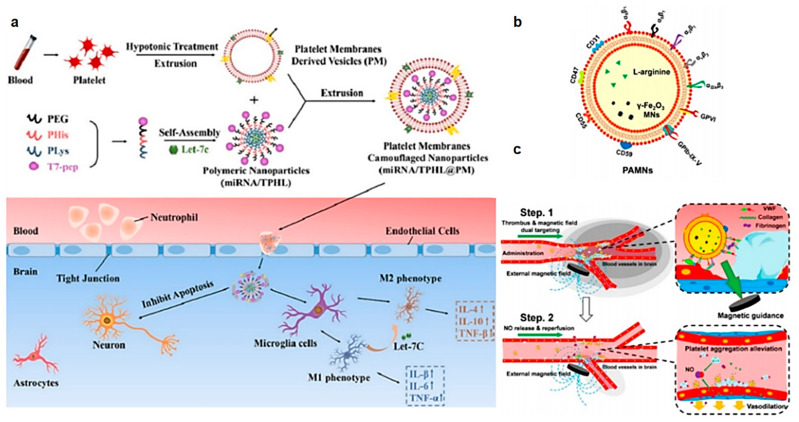
(**a**) Flowchart of miRNA/TPHI@PM preparation and how it functions. Delivery of miRNA through platelet membrane-coated nanoparticles to sites of brain lesions aims to reduce neuronal apoptosis and regulate microglial cell phenotype [217]. Copyright 2022, Elsevier Ltd. (**b**) The structure of PAMNs [219]. Copyright 2021, American Chemical Society. (**c**) Schematic illustration of the therapeutic mechanism of PAMNs. Due to the endowed platelet membrane function and external magnetic targeting, PAMNs rapidly reaches the ischemic region of stroke and achieves rapid targeted delivery of l-arginine. Meanwhile, NO generation in situ allows the induction of vasodilation and the reduction of PLT aggregation [219]. Copyright 2021, American Chemical Society.

A precedent for biomimetic platelet membrane-coated nanoparticles, which mimic platelets and evade the immune system, was established by Zhang et al. in 2015 [193]. Since then, there has been a growing focus on platelet membrane-coated nanoparticles. For example, Cui et al. selected GB, a neuroprotective agent with anti-inflammatory and antioxidant properties, and coated GB with platelet membranes (PM-GB) [216]. Because platelet membranes could target inflammatory injuries, the concentration of GB at the injury site increased, resulting in improved drug delivery to the lesion. The results demonstrated that PM-GB was suitable for the treatment of ISs by inhibiting oxidative stress and reducing iron-related cell death. Zhao et al. designed a platelet membrane-coated nanoparticle with multi-function for the therapy of ISs [217] (Figure 7a). To be specific, when a platelet membrane adheres to inflamed neutrophils, platelet membrane-coated nanoparticles can then follow the neutrophils into the inflamed area. The platelet membrane-coated nanoparticles consisted of T7 peptide, PHis (an acid-responsive fragment), and MiRNA-Let-7c drugs. Based on the multiple functional fragments, this NDDS could be BBB targeting and swell to release miRNA to inhibit M1 cell polarization. Li et al. developed platelet-derived bio-nanobubbles with integrated diagnostic capabilities using platelet vesicles [218]. In addition to platelet vesicles, the formulation also includes γ-Fe_2_O_3_ and the NO precursor (L-arginine). The results showed that it could increase the flow of blood to the lesion site, prolong the treatment window through the vasodilatory effect of NO, and provide auxiliary diagnostic imaging [219] (Figure 7b). After that, Li further conducted the therapeutic mechanism of PAMNs. The results illustrated that it can rapidly dilate vessels and improve vascular flow, which is beneficial for the early-stage therapy of ISs (Figure 7c).

To sum up, the properties of platelets allow for platelet membrane-coated nanoparticles to be widely used for vascular embolism disease [220,221,222,223,224,225,226]. 

#### 3.4.3. WBC Membrane-Coated Nanoparticles

WBCs are important blood cells of the body’s immune system, encompassing various types, such as NK cells, neutrophils, macrophages, and lymphocytes. As the body’s guardians, WBCs protect the body from disease and arrive at the injury site immediately when an IS occurs [227]. In contrast to RBCs and platelets, WBCs have nuclear structures and are less abundant, rendering their membrane isolation relatively challenging. To be noted, natural WBC membranes without modification have the capability of targeting inflammatory sites and tumor tissue [228,229]. 

**Figure 8:**
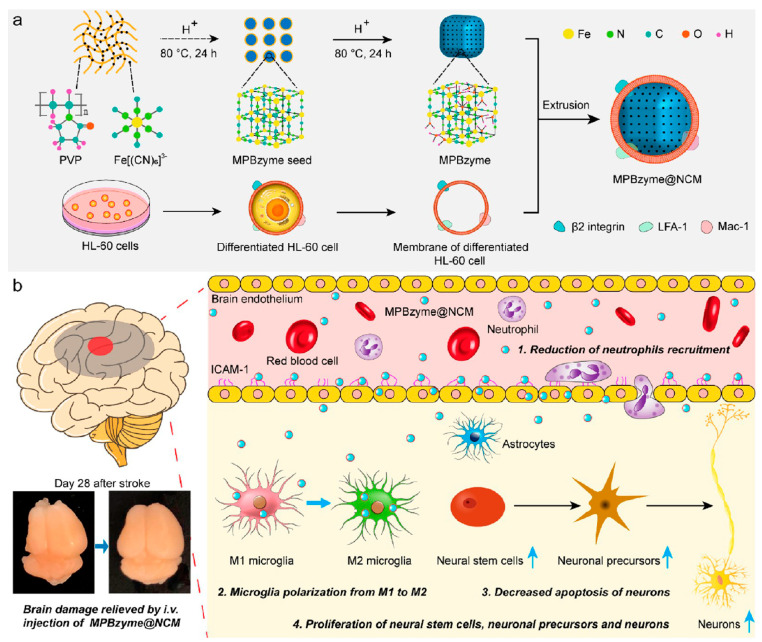
(**a**) Preparation of MPBzyme@NCM; (**b**) The effect of MPBzyme@NCM in vivo. Long-term therapeutic effects of stroke were exerted through the ability to reduce neutrophil recruitment, regulate microglia polarization from M1 to M2, reduce neuronal apoptosis, and increase neural stem cell proliferation [234]. Copyright 2021, American Chemical Society.

Here, we mainly discuss membranes from neutrophils and macrophages to fabricate NDDSs. Neutrophils are virtually absent from the brain, but after an IS occurs, neutrophils rapidly increase in numbers and enter the ischemic lesion area in a short time [230]. The utilization of magnetic probes coated with neutrophil membranes holds promise for imaging neuroinflammation in ISs [231]. Neutrophil membranes or extracellular vesicles can be used to intracerebrally target, both of which have satisfactory effects in ISs [232,233]. Feng et al. treated ISs with neutrophil membrane-encapsulated Prussian blue nanoenzyme (MPBzyme@NCM) [234] (Figure 8a). The results showed that MPBzyme@NCM could polarize microglia from the M1 phenotype to the M2 phenotype, reduce inflammatory responses, and protect injured brain tissue (Figure 8b). This strategy showed promise in extending its potential application to other CNS illnesses.

**Figure 9:**
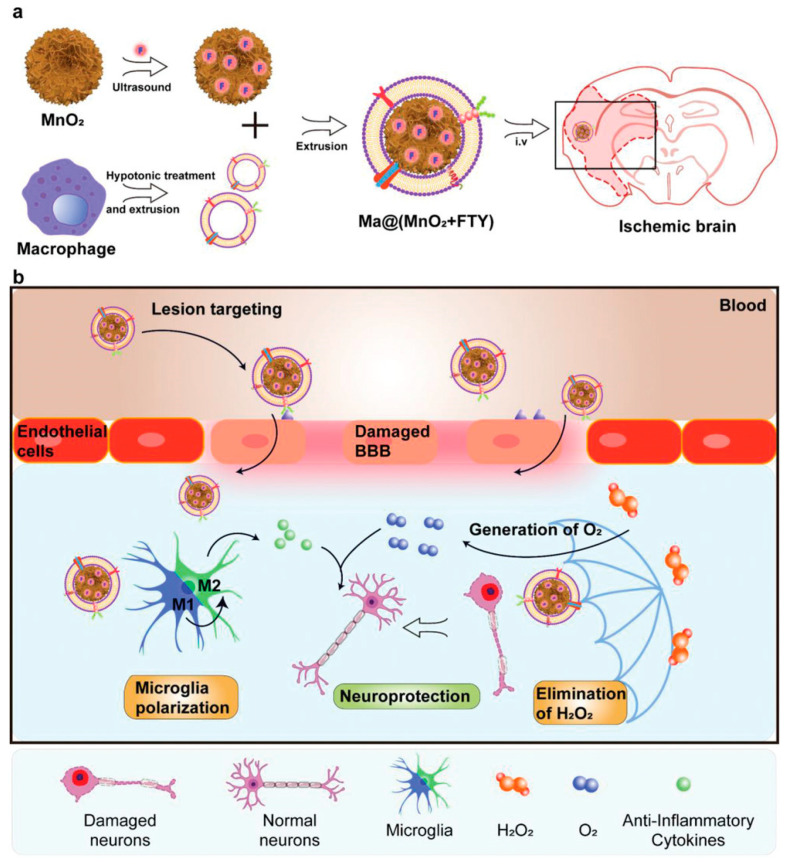
(**a**) Preparation of Ma@(MnO_2_ + FTY); (**b**) Schematic illustration of the effect of Ma@(MnO_2_ + FTY) [52]. Copyright 2021, Wiley-VCH.

Macrophages are immune cells with the capabilities of pathogen recognition and phagocytic clearance. At the onset of an inflammatory response, macrophages exhibit a tendency towards inflammation and migrate to the inflammatory site to phagocytose and eliminate pathogens [235,236]. Based on these remarkable features, macrophage membrane-coated nanoparticles were widely applied in delivery studies [237]. Li et al. developed mesoporous SiO_2_ nanoparticles loaded with the neuroprotectant FTY-720 (MnO_2_ + FTY) and then wrapped MnO_2_ + FTY with macrophage membrane vesicles to treat ISs [52] (Figure 9a). In this system, macrophage membranes confer nanoparticles with the ability to target inflammatory lesions. MnO_2_ nanoparticles have broad surface and CAT properties, which can effectively scavenge excess ROS and promote O_2_ conversion, thereby reducing inflammatory responses and rescuing dying neurons. FTY-720 reverses the pro-inflammatory microenvironment (Figure 9b). Su et al. used macrophage membranes to encapsulate curcumin for treating ISs, yielding promising therapeutic outcomes. This study offers valuable insights in combining the traditional Chinese medicine and modern technology [238].

In conclusion, the utilization of WBC membrane-coated nanoparticles emerges as a highly promising avenue for the treatment of both inflammation and cancer. Despite the significant advancements made in this field, further exploration is warranted to fully harness the capabilities of WBC membrane-coated nanoparticles.

#### 3.4.4. Cancer Cell Membrane-Coated Nanoparticles

**Figure 10:**
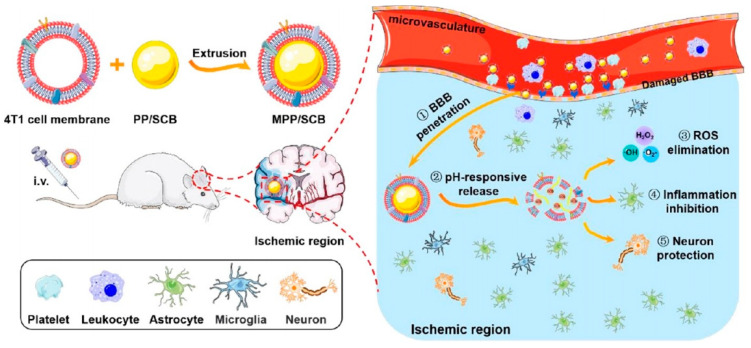
Preparation of MPP/SCB and its effect on ISs [246]. Copyright 2021, American Chemical Society.

Cancer cells exhibit immune evasion and homology-targeting capabilities; thus, nanoparticles encapsulated within cancer cell membranes can be used for targeted therapy [197,239,240]. This strategy has been widely used in cancer homologous-targeted therapy and cancer vaccine development [241,242,243,244,245]. Apart from cancer, He et al. found that cancer cell membrane-coated nanoparticles could be applied to IS treatment. As shown in Figure 10, they developed a novel biomimetic nanoplatform, termed MPP/SCB, by cloaking a succinobucol-loaded pH-sensitive polymeric nanovehicle with a 4T1 cell membrane. They drew inspiration from the BBB-penetrating ability of 4T1 cancer cells during brain metastasis. The primary factors contributing to this phenomenon include the heightened affinity of certain adhesion molecules highly expressed on the membrane of 4T1 cells, enabling adhesion to leukocytes, endothelial cells, and platelets [246]. MPP/SCB significantly improved microvascular reperfusion in the ischemic hemisphere, leading to a remarkable 69.9% reduction in infarct volume and demonstrating superior neuroprotective effects compared to uncamouflaged PP/SCB. Although MPP/SCB show negligible biotoxicity, the potential presence of numerous tumor antigens on cancer cell membranes poses an unknown risk that warrants further investigation and validation. Their findings highlight the potential of cancer cell membrane-coated nanoparticles for the targeted therapy of cerebral ischemic lesions in ISs and inspire us to explore additional functions of cancer cell membranes beyond their interaction with cancer cell homologous targets.

#### 3.4.5. Other Cell Membrane-Coated Nanoparticles

Stem cells, characterized by their capacity for self-renewal and differentiation, exhibit the proficient recognition and repair of damaged tissues, homing ability, inflammation suppression, and tumor-targeting abilities [247]. Stem cell studies in treating ISs are extensive, with a predominant focus on the transplantation of stem cells or the administration of stem cell-derived vesicles and trophic cytokines [248,249,250,251,252,253]. At present, fewer studies have been conducted on stem cell membrane-coated nanoparticles for IS therapy [254]. We notice that there was a study using stem cell membrane-coated nanoparticles as a delivery platform, which leveraged the SDF-1/CXCR4 pathway to enhance targeted drug delivery in ISs [255]. By encapsulating glyburide-loaded PLGA within stem cell membranes, Ma et al. significantly improved stroke treatment efficacy [256]. This innovative approach not only underscores the importance of the SDF-1/CXCR4 axis in cell migration and homing but also offers a promising strategy for enhancing intracerebral drug delivery. 

Bacterial membrane-coated nanoparticles have been broadly used for targeted tumor therapy, antimicrobial therapy, vaccine development, and so on [257,258,259,260,261]. Although it has been reported that anaerobic bacteria can cross the BBB to treat gliomas [262], we did not find any reports of treating ISs with nanoparticles coated with bacterial membranes. 

## 4. Summary and Perspective

ISs are the second leading cause of death in the world, which is attributed to the narrow therapeutic window and the complexity of the disease progression involving multiple mechanisms, including neuro-excitotoxicity, oxidative stress, neuroinflammation, mitochondrial damage, and so on. Understanding how an IS occurs is a necessary prerequisite for attempting to resolve the disease. Thrombolysis is the preferred treatment to prevent neuronal damage. The prognosis after thrombolysis is key to restoring normal limb function. 

With the rapid development of nanotechnology, polymer nanoparticles, inorganic nanoparticles, and liposomes, membrane-coated nanoparticles have gradually emerged in diagnosing and treating numerous diseases, including ISs [263,264,265,266]. Some have successfully transitioned from the laboratory to clinical applications. However, there are still some problems that limit their application. In general, polymers increase drug stability and can be surface modified to enhance targeting or improve biocompatibility. However, the relatively high cost and complex preparation process limit their mass production. Some inorganic nanoparticles exhibit exceptional photothermal and imaging properties, but as exogenous materials, they still encounter challenges related to unknown cytotoxicity and complex degradation issues. Among them, magnetic nanoparticles show promising prospects, and functionalized magnetic nanoparticles are the trend of future development. Additionally, optimizing their multifunctional imaging capabilities holds significant potential for advancing the clinical diagnosis and treatment of diseases [267,268]. Liposomes have notably progressed compared to other nanoparticles, boasting advantages such as low toxicity and high biocompatibility. Various drugs have been successfully delivered using liposomes. However, liposomes are expensive, which can exacerbate the financial burden on patients. Membrane-coated nanoparticles have emerged as a novel approach for targeted drug delivery in recent years. It is characterized by the use of biomimetic membranes, where the drug is camouflaged as an endogenous substance. This strategy helps evade the RES clearance, thereby extending the drug’s half-life. Moreover, membrane-coated nanoparticles tend to exhibit targeting capabilities, enhancing the efficacy of precise therapy. The experimental results have yielded promising outcomes, suggesting a bright future ahead. However, these findings are still in their infancy, necessitating further investments. The translation from the laboratory to clinical settings necessitates the careful consideration of immune rejection, particularly regarding proteins or genes carried by biomembranes sourced from different origins. In addition, the fusion of multiple membranes is emerging as a trend. Challenges such as difficulty in scale-up production, uncertainty in variant proteins in membranes, and ensuring the controllable preparations necessitate ongoing research. Nevertheless, it is undeniable that membrane-coated nanoparticles have inherent advantages and hold broad potential for application.

Overall, an IS is a complex pathophysiological process, in which the interference of the BBB and other factors significantly impacts drug delivery. NDDSs struggle to deliver drugs effectively within the brain. Most studies involving NDDSs are still in the laboratory stage, and the translation of NDDSs into clinical practice remains a daunting challenge. 

## Figures and Tables

**Table 1:** A summary of the key features of various cell membranes.

Cell Membrane Source	Key Features	Function	Reference
Red blood cell (RBC)	Immune evasion Easy to extract Surface expression of CD47	Prolonged circulation	[192]
Platelet	Specific targeting of damaged tissue Adherence to inflammatory neutrophil Surface expression of CD47, CD55 and CD59	Injury sites targeting	[193]
White blood cell (WBC)	Specific targeting of inflammatory tissue Endothelial adherence Adhesion at tumor sites Penetration of the BBB	Tumor and inflammatory site targeting	[194,195,196]
Cancer cell	Immune evasion Homologous targeting Anti-tumor ability	Tumor targeting	[197]
Stem cell	Immune evasion Tumor-specific properties Homing ability	Tumor targeting Inflammatory damage targeting	[198,199]
Bacteria	Promoting adaptive immunity	Tumor anaerobic targeting	[200,201,202]

## References

[1] Sarfo F.S., Mobula L.M., Plange-Rhule J., Ansong D., Ofori-Adjei D. (2018). Incident stroke among Ghanaians with hypertension and diabetes: A multicenter, prospective cohort study. J. Neurol. Sci..

[2] Leys D., Deplanque D., Mounier-Vehier C., Mackowiak-Cordoliani M.A., Lucas C., Bordet R. (2002). Stroke prevention: Management of modifiable vascular risk factors. J. Neurol..

[3] Pandey A., Patel M.R., Willis B., Gao A., Leonard D., Das S.R., Defina L., Berry J.D. (2016). Association between midlife cardiorespiratory fitness and risk of stroke: The cooper center longitudinal study. Stroke.

[4] Alloubani A., Saleh A., Abdelhafiz I. (2018). Hypertension and diabetes mellitus as a predictive risk factors for stroke. Diabetes. Metab. Syndr..

[5] Iadecola C., Buckwalter M.S., Anrather J. (2020). Immune responses to stroke: Mechanisms, modulation, and therapeutic potential. J. Clin. Investig..

[6] Campbell B.C.V., De Silva D.A., Macleod M.R., Coutts S.B., Schwamm L.H., Davis S.M., Donnan G.A. (2019). Ischaemic stroke. Nat. Rev. Dis. Primers..

[7] Bautista-Perez S.M., Silva-Islas C.A., Sandoval-Marquez O.U., Toledo-Toledo J., Bello-Martínez J.M., Barrera-Oviedo D., Maldonado P.D. (2023). Antioxidant and Anti-Inflammatory Effects of Garlic in Ischemic Stroke: Proposal of a New Mechanism of Protection through Regulation of Neuroplasticity. Antioxidants.

[8] Karsy M., Brock A., Guan J., Taussky P., Kalani M.Y., Park M.S. (2017). Neuroprotective strategies and the underlying molecular basis of cerebrovascular stroke. Neurosurg. Focus..

[9] Jayaraj R.L., Azimullah S., Beiram R., Jalal F.Y., Rosenberg G.A. (2019). Neuroinflammation: Friend and foe for ischemic stroke. J. Neuroinflamm..

[10] Wu S., Wu B., Liu M., Chen Z., Wang W., Anderson C.S., Sandercock P., Wang Y., Huang Y., Cui L. (2019). Stroke in China: Advances and challenges in epidemiology, prevention, and management. Lancet Neurol..

[11] Campbell B.C.V., Khatri P. (2020). Stroke. Lancet.

[12] Campbell B.C. (2019). Advances in stroke medicine. Med. J. Aust..

[13] Nogueira R.G., Jadhav A.P., Haussen D.C., Bonafe A., Budzik R.F., Bhuva P., Yavagal D.R., Ribo M., Cognard C., Hanel R.A. (2018). Thrombectomy 6 to 24 hours after stroke with a mismatch between deficit and infarct. N. Engl. J. Med..

[14] Albers G.W., Marks M.P., Kemp S., Christensen S., Tsai J.P., Ortega-Gutierrez S., McTaggart R.A., Torbey M.T., Kim-Tenser M., Leslie-Mazwi T. (2018). Thrombectomy for stroke at 6 to 16 hours with selection by perfusion imaging. N. Engl. J. Med..

[15] Zhang Y., Gao Z., Wang D., Zhang T., Sun B., Mu L., Wang J., Liu Y., Kong Q., Liu X. (2014). Accumulation of natural killer cells in ischemic brain tissues and the chemotactic effect of IP-10. J. Neuroinflamm..

[16] Lattanzi S., Brigo F., Trinka E., Cagnetti C., Di Napoli M., Silvestrini M. (2019). Neutrophil-to-Lymphocyte ratio in acute cerebral hemorrhage: A system review. Transl. Stroke. Res..

[17] Anttila J.E., Whitaker K.W., Wires E.S., Harvey B.K., Airavaara M. (2017). Role of microglia in ischemic focal stroke and recovery: Focus on Toll-like receptors. Prog. Neuropsychopharmacol. Biol. Psychiatry..

[18] Liu X., Liu J., Zhao S., Zhang H., Cai W., Cai M., Ji X., Leak R.K., Gao Y., Chen J. (2016). Interleukin-4 is essential for microglia/macrophage M2 polarization and long-term recovery after cerebral ischemia. Stroke.

[19] Cherry J.D., Olschowka J.A., O’Banion M.K. (2014). Neuroinflammation and M2 microglia: The good, the bad, and the inflamed. J. Neuroinflamm..

[20] Chen J., Jin J., Li K., Shi L., Wen X., Fang F. (2022). Progresses and prospects of neuroprotective agents-loaded nanoparticles and biomimetic material in ischemic stroke. Front. Cell. Neurosci..

[21] Lee X.R., Xiang G.L. (2018). Effects of edaravone, the free radical scavenger, on outcomes in acute cerebral infarction patients treated with ultra-early thrombolysis of recombinant tissue plasminogen activator. Clin. Neurol. Neurosurg..

[22] Xu Y., Chen A., Wu J., Wan Y., You M., Gu X., Guo H., Tan S., He Q., Hu B. (2022). Nanomedicine: An Emerging Novel Therapeutic Strategy for Hemorrhagic Stroke. Int. J. Nanomed..

[23] Parvez S., Kaushik M., Ali M., Alam M.M., Ali J., Tabassum H., Kaushik P. (2022). Dodging blood brain barrier with “nano” warriors: Novel strategy against ischemic stroke. Theranostics.

[24] Bhaskar S., Tian F., Stoeger T., Kreyling W., de la Fuente J.M., Grazú V., Borm P., Estrada G., Ntziachristos V., Razansky D. (2010). Multifunctional nanocarriers for diagnostics, drug delivery and targeted treatment across blood-brain barrier: Perspectives on tracking and neuroimaging. Part Fibre Toxicol..

[25] Santra S., Das S., Sengupta A., Molla M.R. (2023). Tumor acidity-induced surface charge modulation in covalent nanonetworks for activated cellular uptake: Targeted delivery of anticancer drugs and selective cancer cell death. Biomater. Sci..

[26] Peng L., Gineste S., Coudret C., Ciuculescu-Pradines D., Benoît-Marquié F., Mingotaud C., Marty J.-D. (2023). Iron-based hybrid polyionic complexes as chemical reservoirs for the pH-triggered synthesis of Prussian blue nanoparticles. J. Colloid. Interface Sci..

[27] Motta S., Siani P., Donadoni E., Frigerio G., Bonati L., Di Valentin C. (2023). Metadynamics simulations for the investigation of drug loading on functionalized inorganic nanoparticles. Nanoscale.

[28] Grebinyk A., Prylutska S., Grebinyk S., Ponomarenko S., Virych P., Chumachenko V., Kutsevol N., Prylutskyy Y., Ritter U., Frohme M. (2022). Drug delivery with a pH-sensitive star-like dextran-graft polyacrylamide copolymer. Nanoscale. Adv..

[29] Cupaioli F.A., Zucca F.A., Boraschi D., Zecca L. (2014). Engineered nanoparticles. How brain friendly is this new guest?. Prog. Neurobiol..

[30] Dong X., Gao J., Su Y., Wang Z. (2020). Nanomedicine for ischemic stroke. Int. J. Mol. Sci..

[31] Ye C., Zhang W., Zhao Y., Zhang K., Hou W., Chen M., Lu J., Wu J., He R., Gao W. (2022). Prussian blue nanozyme normalizes microenvironment to delay osteoporosis. Adv. Healthc. Mater..

[32] Jin R., Yang G., Li G. (2010). Inflammatory mechanisms in ischemic stroke: Role of inflammatory cells. J. Leukoc. Biol..

[33] Grønberg N.V., Johansen F.F., Kristiansen U., Hasseldam H. (2013). Leukocyte infiltration in experimental stroke. J. Neuroinflamm..

[34] Kim J.Y., Kawabori M., Yenari M.A. (2014). Innate inflammatory responses in stroke: Mechanisms and potential therapeutic targets. Curr. Med. Chem..

[35] Xie C., Liao J., Zhang N., Sun Y., Li Y., Xiong L., Zhang Y., Liu X., Su W., Chen H. (2024). Advanced nano drug delivery systems for neuroprotection against ischemic stroke. Chin. Chem. Lett..

[36] Gong Z., Guo J., Liu B., Guo Y., Cheng C., Jiang Y., Liang N., Hu M., Song T., Yang L. (2023). Mechanisms of immune response and cell death in ischemic stroke and their regulation by natural compounds. Front. Immunol..

[37] Andrabi S.S., Parvez S., Tabassum H. (2020). Ischemic stroke and mitochondria: Mechanisms and targets. Protoplasma.

[38] Chamorro Á., Dirnagl U., Urra X., Planas A.M. (2016). Neuroprotection in acute stroke: Targeting excitotoxicity, oxidative and nitrosative stress, and inflammation. Lancet Neurol..

[39] Anrather J., Iadecola C. (2016). Inflammation and stroke: An overview. Neurotherapeutics.

[40] Tuo Q.Z., Zhang S.T., Lei P. (2022). Mechanisms of neuronal cell death in ischemic stroke and their therapeutic implications. Med. Res. Rev..

[41] Jurcau A., Simion A. (2021). Neuroinflammation in cerebral ischemia and ischemia/reperfusion injuries: From pathophysiology to therapeutic strategies. Int. J. Mol. Sci..

[42] Lai T.W., Zhang S., Wang Y.T. (2014). Excitotoxicity and stroke: Identifying novel targets for neuroprotection. Prog. Neurobiol..

[43] Mittal M., Siddiqui M.R., Tran K., Reddy S.P., Malik A.B. (2014). Reactive oxygen species in inflammation and tissue injury. Antioxid. Redox Signal..

[44] Allen C.L., Bayraktutan U. (2009). Oxidative stress and its role in the pathogenesis of ischaemic stroke. Int. J. Stroke.

[45] He W., Zhang Z., Sha X. (2021). Nanoparticles-mediated emerging approaches for effective treatment of ischemic stroke. Biomaterials.

[46] Akbik F., Xu H., Xian Y., Shah S., Smith E.E., Bhatt D.L., Matsouaka R.A., Fonarow G.C., Schwamm L.H. (2020). Trends in Reperfusion Therapy for In-Hospital Ischemic Stroke in the Endovascular Therapy Era. JAMA Neurol..

[47] Bai J., Lyden P.D. (2015). Revisiting cerebral postischemic reperfusion injury: New insights in understanding reperfusion failure, hemorrhage, and edema. Int. J. Stroke.

[48] Deng Y.H., He H.Y., Yang L.Q., Zhang P.Y. (2016). Dynamic changes in neuronal autophagy and apoptosis in the ischemic penumbra following permanent ischemic stroke. Neural Regen. Res..

[49] Wang X., Fang Y., Huang Q., Xu P., Lenahan C., Lu J., Zheng J., Dong X., Shao A., Zhang J. (2021). An updated review of autophagy in ischemic stroke: From mechanisms to therapies. Exp. Neurol..

[50] Seners P., Baron J.C., Olivot J.M., Albers G.W. (2024). Does imaging of the ischemic penumbra have value in acute ischemic stroke with large vessel occlusion?. Curr. Opin. Neurol..

[51] Ya J., Pellumbaj J., Hashmat A., Bayraktutan U. (2024). The role of stem cells as therapeutics for ischaemic stroke. Cells.

[52] Li C., Zhao Z., Luo Y., Ning T., Liu P., Chen Q., Chu Y., Guo Q., Zhang Y., Zhou W. (2021). Macrophage-disguised manganese dioxide nanoparticles for neuroprotection by reducing oxidative stress and modulating inflammatory microenvironment in acute ischemic stroke. Adv. Sci..

[53] Chen H., He Y., Chen S., Qi S., Shen J. (2020). Therapeutic targets of oxidative/nitrosative stress and neuroinflammation in ischemic stroke: Applications for natural product efficacy with omics and systemic biology. Pharmacol. Res..

[54] Zhang H., Feng Y., Si Y., Lu C., Wang J., Wang S., Li L., Xie W., Yue Z., Yong J. (2024). Shank3 ameliorates neuronal injury after cerebral ischemia/reperfusion via inhibiting oxidative stress and inflammation. Redox. Biol..

[55] Li Y., Wang Y., Yang W., Wu Z., Ma D., Sun J., Tao H., Ye Q., Liu J., Ma Z. (2023). ROS-responsive exogenous functional mitochondria can rescue neural cells post-ischemic stroke. Front. Cell. Dev. Biol..

[56] Manickam D.S. (2022). Delivery of mitochondria via extracellular vesicles—A new horizon in drug delivery. J. Control. Release.

[57] Dave K.M., Stolz D.B., Manickam D.S. (2023). Delivery of mitochondria-containing extracellular vesicles to the BBB for ischemic stroke therapy. Expert. Opin. Drug. Deliv..

[58] Kostandy B.B. (2012). The role of glutamate in neuronal ischemic injury: The role of spark in fire. Neurol. Sci..

[59] Mongin A.A. (2007). Disruption of ionic and cell volume homeostasis in cerebral ischemia: The perfect storm. Pathophysiology.

[60] Hu H.J., Song M. (2017). Disrupted Ionic homeostasis in ischemic stroke and new therapeutic targets. J. Stroke Cerebrovasc. Dis..

[61] Bading H. (2017). Therapeutic targeting of the pathological triad of extrasynaptic NMDA receptor signaling in neurodegenerations. J. Exp. Med..

[62] Huo Y., Feng X., Niu M., Wang L., Xie Y., Wang L., Ha J., Cheng X., Gao Z., Sun Y. (2021). Therapeutic time windows of compounds against NMDA receptors signaling pathways for ischemic stroke. J. Neurosci. Res..

[63] Winterbourn C.C. (2008). Reconciling the chemistry and biology of reactive oxygen species. Nat. Chem. Biol..

[64] Liu Y., Ai K., Ji X., Askhatova D., Du R., Lu L., Shi J. (2017). Comprehensive insights into the multi-antioxidative mechanisms of melanin nanoparticles and their application to protect brain from injury in ischemic stroke. J. Am. Chem. Soc..

[65] Zhang K., Tu M., Gao W., Cai X., Song F., Chen Z., Zhang Q., Wang J., Jin C., Shi J. (2019). Hollow prussian blue nanozymes drive neuroprotection against ischemic stroke via attenuating oxidative stress, counteracting inflammation, and suppressing cell apoptosis. Nano Lett..

[66] Faiz M., Sachewsky N., Gascón S., Bang K.W.A., Morshead C.M., Nagy A. (2015). Adult neural stem cells from the subventricular zone give rise to reactive astrocytes in the cortex after stroke. Cell Stem Cell.

[67] Mayor D., Tymianski M. (2018). Neurotransmitters in the mediation of cerebral ischemic injury. Neuropharmacology.

[68] Wang F., Xie X., Xing X., Sun X. (2022). Excitatory Synaptic Transmission in Ischemic Stroke: A New Outlet for Classical Neuroprotective Strategies. Int. J. Mol. Sci..

[69] Liu H., Wu X., Luo J., Zhao L., Li X., Guo H., Bai H., Cui W., Guo W., Feng D. (2020). Adiponectin peptide alleviates oxidative stress and NLRP3 inflammasome activation after cerebral ischemia-reperfusion injury by regulating AMPK/GSK-3β. Exp. Neurol..

[70] Orellana-Urzúa S., Rojas I., Líbano L., Rodrigo R. (2020). Pathophysiology of Ischemic Stroke: Role of Oxidative Stress. Curr. Pharm. Des..

[71] Rodrigo R., Fernández-Gajardo R., Gutiérrez R., Matamala J.M., Carrasco R., Miranda-Merchak A., Feuerhake W. (2013). Oxidative stress and pathophysiology of ischemic stroke: Novel therapeutic opportunities. CNS Neurol. Disord-Drug Targets.

[72] Orellana-Urzúa S., Briones-Valdivieso C., Chichiarelli S., Saso L., Rodrigo R. (2023). Potential role of natural antioxidants in countering reperfusion injury in acute myocardial infarction and ischemic stroke. Antioxidants.

[73] Salatin S., Farhoudi M., Farjami A., Maleki Dizaj S., Sharifi S., Shahi S. (2023). Nanoparticle formulations of antioxidants for the management of oxidative stress in stroke: A review. Biomedicines.

[74] Lakhan S.E., Kirchgessner A., Hofer M. (2009). Inflammatory mechanisms in ischemic stroke: Therapeutic approaches. J. Transl. Med..

[75] Zhang W., Tian T., Gong S.X., Huang W.Q., Zhou Q.Y., Wang A.P., Tian Y. (2021). Microglia-associated neuroinflammation is a potential therapeutic target for ischemic stroke. Neural Regen. Res..

[76] Greenhalgh A.D., David S., Bennett F.C. (2020). Immune cell regulation of glia during CNS injury and disease. Nat. Rev. Neurosci..

[77] Maida C.D., Norrito R.L., Daidone M., Tuttolomondo A., Pinto A. (2020). Neuroinflammatory mechanisms in ischemic stroke: Focus on cardioembolic stroke, background, and therapeutic approaches. Int. J. Mol. Sci..

[78] Ma Y., Wang J., Wang Y., Yang G.Y. (2017). The biphasic function of microglia in ischemic stroke. Prog. Neurobiol..

[79] Li F., Ma Q., Zhao H., Wang R., Tao Z., Fan Z., Zhang S., Li G., Luo Y. (2018). L-3-n-Butylphthalide reduces ischemic stroke injury and increases M2 microglial polarization. Metab. Brain. Dis..

[80] Kronenberg G., Uhlemann R., Richter N., Klempin F., Wegner S., Staerck L., Wolf S., Uckert W., Kettenmann H., Endres M. (2018). Distinguishing features of microglia- and monocyte-derived macrophages after stroke. Acta Neuropathol..

[81] Guruswamy R., ElAli A. (2017). Complex roles of microglial cells in ischemic stroke pathobiology: New insights and future directions. Int. J. Mol. Sci..

[82] Vay S.U., Flitsch L.J., Rabenstein M., Rogall R., Blaschke S., Kleinhaus J., Reinert N., Bach A., Fink G.R., Schroeter M. (2018). The plasticity of primary microglia and their multifaceted effects on endogenous neural stem cells in vitro and in vivo. J. Neuroinflamm..

[83] Ye Y., Jin T., Zhang X., Zeng Z., Ye B., Wang J., Zhong Y., Xiong X., Gu L. (2019). Meisoindigo protects against focal cerebral ischemia-reperfusion injury by inhibiting NLRP3 inflammasome activation and regulating microglia/macrophage polarization via TLR4/NF-κB signaling pathway. Front. Cell. Neurosci..

[84] Kanazawa M., Miura M., Toriyabe M., Koyama M., Hatakeyama M., Ishikawa M., Nakajima T., Onodera O., Takahashi T., Nishizawa M. (2017). Microglia preconditioned by oxygen-glucose deprivation promote functional recovery in ischemic rats. Sci. Rep..

[85] Tang Y., Le W. (2016). Differential roles of M1 and M2 microglia in neurodegenerative diseases. Mol. Neurobiol..

[86] Fu Y., Liu Q., Anrather J., Shi F.D. (2015). Immune interventions in stroke. Nat. Rev. Neurol..

[87] Gan Y., Liu Q., Wu W., Yin J.X., Bai X.F., Shen R., Wang Y., Chen J., La Cava A., Poursine-Laurent J. (2014). Ischemic neurons recruit natural killer cells that accelerate brain infarction. Proc. Natl. Acad. Sci. USA.

[88] Doyle K.P., Simon R.P., Stenzel-Poore M.P. (2008). Mechanisms of ischemic brain damage. Neuropharmacology.

[89] Phipps M.S., Cronin C.A. (2020). Management of acute ischemic stroke. Brit. Med. J..

[90] Mitchell M.J., Billingsley M.M., Haley R.M., Wechsler M.E., Peppas N.A., Langer R. (2021). Engineering precision nanoparticles for drug delivery. Nat. Rev. Drug. Discov..

[91] Furtado D., Björnmalm M., Ayton S., Bush A.I., Kempe K., Caruso F. (2018). Overcoming the Blood-Brain Barrier: The Role of Nanomaterials in Treating Neurological Diseases. Adv. Mater..

[92] Tian X., Fan T., Zhao W., Abbas G., Han B., Zhang K., Li N., Liu N., Liang W., Huang H. (2021). Recent advances in the development of nanomedicines for the treatment of ischemic stroke. Bioact. Mater..

[93] Stoll G., Bendszus M. (2010). New approaches to neuroimaging of central nervous system inflammation. Curr. Opin. Neurol..

[94] Qiao R., Fu C., Forgham H., Javed I., Huang X., Zhu J., Whittaker A.K., Davis T.P. (2023). Magnetic iron oxide nanoparticles for brain imaging and drug delivery. Adv. Drug. Deliv. Rev..

[95] Tang L., Fu C., Zhang A., Li X., Cao Y., Feng J., Liu H., Dong H., Wang W. (2023). Harnessing nanobiotechnology for cerebral ischemic stroke management. Biomater. Sci..

[96] Chen W., Jiang L., Hu Y., Fang G., Yang B., Li J., Liang N., Wu L., Hussain Z. (2021). Nanomedicines, an emerging therapeutic regimen for treatment of ischemic cerebral stroke: A review. J. Control. Release.

[97] Fournier E., Passirani C., Montero-Menei C.N., Benoit J.P. (2003). Biocompatibility of implantable synthetic polymeric drug carriers: Focus on brain biocompatibility. Biomaterials.

[98] Miura Y., Takenaka T., Toh K., Wu S., Nishihara H., Kano M.R., Ino Y., Nomoto T., Matsumoto Y., Koyama H. (2013). Cyclic RGD-linked polymeric micelles for targeted delivery of platinum anticancer drugs to glioblastoma through the blood-brain tumor barrier. ACS Nano.

[99] Kassem S., Piletsky S.S., Yesilkaya H., Gazioglu O., Habtom M., Canfarotta F., Piletska E., Spivey A.C., Aboagye E.O., Piletsky S.A. (2022). Assessing the in vivo biocompatibility of molecularly imprinted polymer nanoparticles. Polymers.

[100] Saraiva C., Praça C., Ferreira R., Santos T., Ferreira L., Bernardino L. (2016). Nanoparticle-mediated brain drug delivery: Overcoming blood-brain barrier to treat neurodegenerative diseases. J. Control. Release.

[101] Chen L., Gao X. (2017). The application of nanoparticles for neuroprotection in acute ischemic stroke. Ther. Deliv..

[102] Zhou J., Patel T.R., Sirianni R.W., Strohbehn G., Zheng M.Q., Duong N., Schafbauer T., Huttner A.J., Huang Y., Carson R.E. (2013). Highly penetrative, drug-loaded nanocarriers improve treatment of glioblastoma. Proc. Natl. Acad. Sci. USA.

[103] Cooke M.J., Wang Y., Morshead C.M., Shoichet M.S. (2011). Controlled epi-cortical delivery of epidermal growth factor for the stimulation of endogenous neural stem cell proliferation in stroke-injured brain. Biomaterials.

[104] Wang Y., Cooke M.J., Morshead C.M., Shoichet M.S. (2012). Hydrogel delivery of erythropoietin to the brain for endogenous stem cell stimulation after stroke injury. Biomaterials.

[105] Wang Y., Cooke M.J., Sachewsky N., Morshead C.M., Shoichet M.S. (2013). Bioengineered sequential growth factor delivery stimulates brain tissue regeneration after stroke. J. Control. Release.

[106] Song Z., Fang J.-h., Wang Z., Xiao R., Guo X., Zhou S. (2023). Rod-shaped polymeric nanoparticles intervene neutrophils for efficient ischemic stroke therapy. Adv. Funct. Mater..

[107] Grayston A., Zhang Y., Garcia-Gabilondo M., Arrúe M., Martin A., Kopcansky P., Timko M., Kovac J., Strbak O., Castellote L. (2021). Endovascular administration of magnetized nanocarriers targeting brain delivery after stroke. J. Cereb. Blood Flow Metab..

[108] Suk J.S., Xu Q., Kim N., Hanes J., Ensign L.M. (2016). PEGylation as a strategy for improving nanoparticle-based drug and gene delivery. Adv. Drug Deliv. Rev..

[109] Zhang L., Han Y., Wu X., Chen B., Liu S., Huang J., Kong L., Wang G., Ye Z. (2023). Research progress on the mechanism of curcumin in cerebral ischemia/reperfusion injury: A narrative review. Apoptosis.

[110] Wang Y., Luo J., Li S.Y. (2019). Nano-Curcumin simultaneously protects the Blood-Brain Barrier and reduces M1 microglial activation during cerebral ischemia-reperfusion injury. ACS Appl. Mater. Interfaces.

[111] Cheng Y., Cheng A., Jia Y., Yang L., Ning Y., Xu L., Zhong Y., Zhuang Z., Guan J., Zhang X. (2021). pH-Responsive multifunctional theranostic rapamycin-loaded nanoparticles for imaging and treatment of acute ischemic stroke. ACS Appl. Mater. Interfaces.

[112] Nan D., Jin H., Yang D., Yu W., Jia J., Yu Z., Tan H., Sun Y., Hao H., Qu X. (2021). Combination of polyethylene glycol-conjugated urokinase nanogels and urokinase for acute ischemic stroke therapeutic implications. Transl. Stroke. Res..

[113] Kalomiraki M., Thermos K., Chaniotakis N.A. (2016). Dendrimers as tunable vectors of drug delivery systems and biomedical and ocular applications. Int. J. Nanomed..

[114] Wang J., Li B., Qiu L., Qiao X., Yang H. (2022). Dendrimer-based drug delivery systems: History, challenges, and latest developments. J. Biol. Eng..

[115] Shaikh A., Kesharwani P., Gajbhiye V. (2022). Dendrimer as a momentous tool in tissue engineering and regenerative medicine. J. Control. Release.

[116] Arkas M., Vardavoulias M., Kythreoti G., Giannakoudakis D.A. (2023). Dendritic polymers in tissue engineering: Contributions of PAMAM, PPI PEG and PEI to injury restoration and bioactive scaffold evolution. Pharmaceutics.

[117] Zhang F., Trent Magruder J., Lin Y.A., Crawford T.C., Grimm J.C., Sciortino C.M., Wilson M.A., Blue M.E., Kannan S., Johnston M.V. (2017). Generation-6 hydroxyl PAMAM dendrimers improve CNS penetration from intravenous administration in a large animal brain injury model. J. Control. Release.

[118] Sharma A., Porterfield J.E., Smith E., Sharma R., Kannan S., Kannan R.M. (2018). Effect of mannose targeting of hydroxyl PAMAM dendrimers on cellular and organ biodistribution in a neonatal brain injury model. J. Control. Release.

[119] Li X., Naeem A., Xiao S., Hu L., Zhang J., Zheng Q. (2022). Safety challenges and application strategies for the use of Dendrimers in medicine. Pharmaceutics.

[120] Luong D., Kesharwani P., Deshmukh R., Mohd Amin M.C.I., Gupta U., Greish K., Iyer A.K. (2016). PEGylated PAMAM dendrimers: Enhancing efficacy and mitigating toxicity for effective anticancer drug and gene delivery. Acta Biomater..

[121] Luong D., Sau S., Kesharwani P., Iyer A.K. (2017). Polyvalent Folate-Dendrimer-Coated Iron Oxide Theranostic Nanoparticles for Simultaneous Magnetic Resonance Imaging and Precise Cancer Cell Targeting. Biomacromolecules.

[122] Li X., Ta W., Hua R., Song J., Lu W. (2022). A Review on Increasing the Targeting of PAMAM as Carriers in Glioma Therapy. Biomedicines.

[123] Amreddy N., Babu A., Panneerselvam J., Srivastava A., Muralidharan R., Chen A., Zhao Y.D., Munshi A., Ramesh R. (2018). Chemo-biologic combinatorial drug delivery using folate receptor-targeted dendrimer nanoparticles for lung cancer treatment. Nanomedicine.

[124] Sharma R., Kim S.Y., Sharma A., Zhang Z., Kambhampati S.P., Kannan S., Kannan R.M. (2017). Activated Microglia Targeting Dendrimer-Minocycline Conjugate as Therapeutics for Neuroinflammation. Bioconjug Chem..

[125] Sharma R., Kambhampati S.P., Zhang Z., Sharma A., Chen S., Duh E.I., Kannan S., Tso M.O.M., Kannan R.M. (2020). Dendrimer mediated targeted delivery of sinomenine for the treatment of acute neuroinflammation in traumatic brain injury. J. Control. Release.

[126] Jeon P., Choi M., Oh J., Lee M. (2015). Dexamethasone-Conjugated Polyamidoamine Dendrimer for Delivery of the Heme Oxygenase-1 Gene into the Ischemic Brain. Macromol. Biosci..

[127] Santos S.D., Xavier M., Leite D.M., Moreira D.A., Custódio B., Torrado M., Castro R., Leiro V., Rodrigues J., Tomás H. (2018). PAMAM dendrimers: Blood-brain barrier transport and neuronal uptake after focal brain ischemia. J. Control. Release.

[128] Zhang S., Peng B., Chen Z., Yu J., Deng G., Bao Y., Ma C., Du F., Sheu W.C., Kimberly W.T. (2022). Brain-targeting, acid-responsive antioxidant nanoparticles for stroke treatment and drug delivery. Bioact. Mater..

[129] Yu Y., Cheng Y., Tong J., Zhang L., Wei Y., Tian M. (2021). Recent advances in thermo-sensitive hydrogels for drug delivery. J. Mater. Chem. B.

[130] Chen C.Y., Kim T.H., Wu W.C., Huang C.M., Wei H., Mount C.W., Tian Y., Jang S.H., Pun S.H., Jen A.K. (2013). pH-dependent, thermosensitive polymeric nanocarriers for drug delivery to solid tumors. Biomaterials.

[131] Zhang X., Zhou J., Gu Z., Zhang H., Gong Q., Luo K. (2021). Advances in nanomedicines for diagnosis of central nervous system disorders. Biomaterials.

[132] Giner-Casares J.J., Henriksen-Lacey M., Coronado-Puchau M., Liz-Marzán L.M. (2016). Inorganic nanoparticles for biomedicine: Where materials scientists meet medical research. Mater. Today.

[133] Bigham N.P., Wilson J.J. (2023). Metal coordination complexes as therapeutic agents for ischemia-reperfusion injury. J. Am. Chem. Soc..

[134] Bansal S.A., Kumar V., Karimi J., Singh A.P., Kumar S. (2020). Role of gold nanoparticles in advanced biomedical applications. Nanoscale Adv..

[135] Chiang M.C., Yang Y.P., Nicol C.J.B., Wang C.J. (2024). Gold nanoparticles in neurological diseases: A review of neuroprotection. Int. J. Mol. Sci..

[136] Wang C., Lin G., Luan Y., Ding J., Li P.C., Zhao Z., Qian C., Liu G., Ju S., Teng G.J. (2019). HIF-prolyl hydroxylase 2 silencing using siRNA delivered by MRI-visible nanoparticles improves therapy efficacy of transplanted EPCs for ischemic stroke. Biomaterials.

[137] Cha B.G., Jeong H.-G., Kang D.-W., Nam M.-J., Kim C.K., Kim D.Y., Choi I.-Y., Ki S.K., Kim S.I., Han J.h. (2018). Customized lipid-coated magnetic mesoporous silica nanoparticle doped with ceria nanoparticles for theragnosis of intracerebral hemorrhage. Nano Res..

[138] Dong H., Du W., Dong J., Che R., Kong F., Cheng W., Ma M., Gu N., Zhang Y. (2022). Depletable peroxidase-like activity of Fe_3_O_4_ nanozymes accompanied with separate migration of electrons and iron ions. Nat. Commun..

[139] Ai Y., Hu Z.N., Liang X.T., Sun H.B., Xin H., Liang Q.-L. (2021). Recent advances in nanozymes: From matters to bioapplications. Adv. Funct. Mater..

[140] Liu Q., Zhang A., Wang R., Zhang Q., Cui D. (2021). A review on metal- and metal oxide-based nanozymes: Properties, mechanisms, and applications. Nanomicro Lett..

[141] Sang D., Luo X., Liu J. (2023). Biological interaction and imaging of ultrasmall gold nanoparticles. Nanomicro Lett..

[142] Nair L.V., Nair R.V., Shenoy S.J., Thekkuveettil A., Jayasree R.S. (2017). Blood brain barrier permeable gold nanocluster for targeted brain imaging and therapy: An in vitro and in vivo study. J. Mater. Chem. B.

[143] Zhou K., Enkhjargal B., Xie Z., Sun C., Wu L., Malaguit J., Chen S., Tang J., Zhang J., Zhang J.H. (2018). Dihydrolipoic acid inhibits lysosomal rupture and NLRP3 through lysosome-associated membrane protein-1/calcium/calmodulin-dependent protein Kinase II/TAK1 pathways after subarachnoid hemorrhage in rat. Stroke.

[144] Xiao L., Wei F., Zhou Y., Anderson G.J., Frazer D.M., Lim Y.C., Liu T., Xiao Y. (2020). Dihydrolipoic acid-gold nanoclusters regulate microglial polarization and have the potential to alter neurogenesis. Nano Lett..

[145] Riley R.S., Day E.S. (2017). Gold nanoparticle-mediated photothermal therapy: Applications and opportunities for multimodal cancer treatment. Wires. Nanomed. Nanobi..

[146] Rastinehad A.R., Anastos H., Wajswol E., Winoker J.S., Sfakianos J.P., Doppalapudi S.K., Carrick M.R., Knauer C.J., Taouli B., Lewis S.C. (2019). Gold nanoshell-localized photothermal ablation of prostate tumors in a clinical pilot device study. Proc. Natl. Acad. Sci. USA.

[147] Liu Y., Wang X., Li X., Qiao S., Huang G., Hermann D.M., Doeppner T.R., Zeng M., Liu W., Xu G. (2021). A co-doped Fe_3_O_4_ nanozyme shows enhanced reactive oxygen and nitrogen species scavenging activity and ameliorates the deleterious effects of ischemic stroke. ACS Appl. Mater. Interfaces.

[148] Wang Z., Cai Y., Zhang Q., Li J., Lin B., Zhao J., Zhang F., Li Y., Yang X., Lu L. (2022). Upregulating HIF-1α to boost the survival of neural stem cells via functional peptides-complexed MRI-Visible nanomedicine for stroke therapy. Adv. Healthc. Mater..

[149] García-Belda P., Prima-García H., Aliena-Valero A., Castelló-Ruiz M., Ulloa-Navas M.J., Ten-Esteve A., Martí-Bonmatí L., Salom J.B., García-Verdugo J.M., Gil-Perotín S. (2022). Intravenous SPION-labeled adipocyte-derived stem cells targeted to the brain by magnetic attraction in a rat stroke model: An ultrastructural insight into cell fate within the brain. Nanomedicine.

[150] Lin B., Lu L., Wang Y., Zhang Q., Wang Z., Cheng G., Duan X., Zhang F., Xie M., Le H. (2021). Nanomedicine Directs Neuronal Differentiation of Neural Stem Cells via Silencing Long Noncoding RNA for Stroke Therapy. Nano Lett..

[151] Lu X., Zhang Y., Wang L., Li G., Gao J., Wang Y. (2021). Development of L-carnosine functionalized iron oxide nanoparticles loaded with dexamethasone for simultaneous therapeutic potential of blood brain barrier crossing and ischemic stroke treatment. Drug Deliv..

[152] Lu Y., Xu Y.J., Zhang G.B., Ling D., Wang M.Q., Zhou Y., Wu Y.D., Wu T., Hackett M.J., Hyo Kim B. (2017). Iron oxide nanoclusters for *T*_1_ magnetic resonance imaging of non-human primates. Nat. Biomed. Eng..

[153] Dadfar S.M., Camozzi D., Darguzyte M., Roemhild K., Varvarà P., Metselaar J., Banala S., Straub M., Güvener N., Engelmann U. (2020). Size-isolation of superparamagnetic iron oxide nanoparticles improves MRI, MPI and hyperthermia performance. J. Nanobiotechnol..

[154] Nowak-Jary J., Machnicka B. (2023). In vivo biodistribution and clearance of magnetic iron oxide nanoparticles for medical applications. Int. J. Nanomed..

[155] Yan Y., Liu Y., Li T., Liang Q., Thakur A., Zhang K., Liu W., Xu Z., Xu Y. (2023). Functional roles of magnetic nanoparticles for the identification of metastatic lymph nodes in cancer patients. J. Nanobiotechnol..

[156] Wang S., He H., Mao Y., Zhang Y., Gu N. (2024). Advances in atherosclerosis theranostics harnessing iron oxide-based nanoparticles. Adv. Sci..

[157] Si G., Du Y., Tang P., Ma G., Jia Z., Zhou X., Mu D., Shen Y., Lu Y., Mao Y. (2024). Unveiling the next generation of MRI contrast agents: Current insights and perspectives on ferumoxytol-enhanced MRI. Natl. Sci. Rev..

[158] Kang D.-W., Kim C.K., Jeong H.-G., Soh M., Kim T., Choi I.-Y., Ki S.-K., Kim D.Y., Yang W., Hyeon T. (2017). Biocompatible custom ceria nanoparticles against reactive oxygen species resolve acute inflammatory reaction after intracerebral hemorrhage. Nano Res..

[159] Goujon G., Baldim V., Roques C., Bia N., Seguin J., Palmier B., Graillot A., Loubat C., Mignet N., Margaill I. (2021). Antioxidant Activity and Toxicity Study of Cerium Oxide Nanoparticles Stabilized with Innovative Functional Copolymers. Adv. Healthc. Mater..

[160] Li X., Han Z., Wang T., Ma C., Li H., Lei H., Yang Y., Wang Y., Pei Z., Liu Z. (2022). Cerium oxide nanoparticles with antioxidative neurorestoration for ischemic stroke. Biomaterials.

[161] He L., Huang G., Liu H., Sang C., Liu X., Chen T. (2020). Highly bioactive zeolitic imidazolate framework-8-capped nanotherapeutics for efficient reversal of reperfusion-induced injury in ischemic stroke. Sci. Adv..

[162] Liao J., Li Y., Fan L., Sun Y., Gu Z., Xu Q.Q., Wang Y., Xiong L., Xiao K., Chen Z.S. (2024). Bioactive ceria nanoenzymes target mitochondria in reperfusion injury to treat ischemic stroke. ACS Nano.

[163] Huang G., Zang J., He L., Zhu H., Huang J., Yuan Z., Chen T., Xu A. (2022). Bioactive nanoenzyme reverses oxidative damage and endoplasmic reticulum stress in neurons under ischemic stroke. ACS Nano.

[164] Zhang K., Qi C., Cai K. (2023). Manganese-based tumor immunotherapy. Adv. Mater..

[165] Bangham A.D., Standish M.M., Watkins J.C. (1965). Diffusion of univalent ions across the lamellae of swollen phospholipids. J. Mol. Biol..

[166] Bruch G.E., Fernandes L.F., Bassi B.L.T., Alves M.T.R., Pereira I.O., Frézard F., Massensini A.R. (2019). Liposomes for drug delivery in stroke. Brain Res. Bull..

[167] Zylberberg C., Matosevic S. (2016). Pharmaceutical liposomal drug delivery: A review of new delivery systems and a look at the regulatory landscape. Drug Deliv..

[168] Liu P., Chen G., Zhang J. (2022). A review of liposomes as a drug delivery system: Current status of approved products, regulatory environments, and future perspectives. Molecules.

[169] So P.W., Ekonomou A., Galley K., Brody L., Sahuri-Arisoylu M., Rattray I., Cash D., Bell J.D. (2019). Intraperitoneal delivery of acetate-encapsulated liposomal nanoparticles for neuroprotection of the penumbra in a rat model of ischemic stroke. Int. J. Nanomed..

[170] Al-Ahmady Z.S., Jasim D., Ahmad S.S., Wong R., Haley M., Coutts G., Schiessl I., Allan S.M., Kostarelos K. (2019). Selective liposomal transport through blood brain barrier disruption in ischemic stroke reveals two distinct therapeutic opportunities. ACS Nano.

[171] Gajbhiye K.R., Pawar A., Mahadik K.R., Gajbhiye V. (2020). PEGylated nanocarriers: A promising tool for targeted delivery to the brain. Colloid. Surf. B..

[172] Thomas R.G., Kim J.H., Kim J.H., Yoon J., Choi K.H., Jeong Y.Y. (2023). Treatment of ischemic stroke by atorvastatin-loaded PEGylated liposome. Transl. Stroke Res..

[173] Ramos-Cabrer P., Agulla J., Argibay B., Pérez-Mato M., Castillo J. (2011). Serial MRI study of the enhanced therapeutic effects of liposome-encapsulated citicoline in cerebral ischemia. Int. J. Pharm..

[174] Zhao Y., Xin Z., Li N., Chang S., Chen Y., Geng L., Chang H., Shi H., Chang Y.Z. (2018). Nano-liposomes of lycopene reduces ischemic brain damage in rodents by regulating iron metabolism. Free. Radic. Biol. Med..

[175] Ishii T., Asai T., Oyama D., Agato Y., Yasuda N., Fukuta T., Shimizu K., Minamino T., Oku N. (2013). Treatment of cerebral ischemia-reperfusion injury with PEGylated liposomes encapsulating FK506. FASEB J..

[176] Koudelka S., Mikulik R., Mašek J., Raška M., Turánek Knotigová P., Miller A.D., Turánek J. (2016). Liposomal nanocarriers for plasminogen activators. J. Control. Release.

[177] Chen Z.L., Huang M., Wang X.R., Fu J., Han M., Shen Y.Q., Xia Z., Gao J.Q. (2016). Transferrin-modified liposome promotes α-mangostin to penetrate the blood-brain barrier. Nanomedicine.

[178] Kariolis M.S., Wells R.C., Getz J.A., Kwan W., Mahon C.S., Tong R., Kim D.J., Srivastava A., Bedard C., Henne K.R. (2020). Brain delivery of therapeutic proteins using an Fc fragment blood-brain barrier transport vehicle in mice and monkeys. Sci. Transl. Med..

[179] Bozzuto G., Molinari A. (2015). Liposomes as nanomedical devices. Int. J. Nanomed..

[180] Sun S., Lv W., Li S., Zhang Q., He W., Min Z., Teng C., Chen Y., Liu L., Yin J. (2023). Smart liposomal nanocarrier enhanced the treatment of ischemic stroke through neutrophil extracellular traps and cyclic guanosine monophosphate-adenosine monophosphate synthase-stimulator of interferon genes (cGAS-STING) pathway inhibition of ischemic penumbra. ACS Nano.

[181] Li Y., Zhang M., Li S., Zhang L., Kim J., Qiu Q., Lu W., Wang J. (2023). Selective ischemic-hemisphere targeting Ginkgolide B liposomes with improved solubility and therapeutic efficacy for cerebral ischemia-reperfusion injury. Asian J. Pharm. Sci..

[182] Yao S., He C., Yuan P., Xu X., Zhou X., Shen L., Hu Q., Slater N.K.H., Sun W., Shen Y. (2023). Real-time objective evaluation of the ischemic stroke through pH-responsive fluorescence imaging. Adv. Healthc. Mater..

[183] Abou-Taleb H.A., Aldosari B.N., Zaki R.M., Afzal O., Tulbah A.S., Shahataa M.G., Abo El-Ela F.I., Salem H.F., Fouad A.G. (2023). Formulation and therapeutic evaluation of isoxsuprine-loaded nanoparticles against diabetes-associated stroke. Pharmaceutics.

[184] Yu S., Li D., Shi A., Long Y., Deng J., Ma Y., Li X., Wen J., Hu Y., He X. (2023). Multidrug-loaded liposomes prevent ischemic stroke through intranasal administration. Biomed. Pharmacother..

[185] Jiang Y., Li W., Wang Z., Lu J. (2023). Lipid-based nanotechnology: Liposome. Pharmaceutics.

[186] Han X., Gong C., Yang Q., Zheng K., Wang Z., Zhang W. (2024). Biomimetic nano-drug delivery system: An emerging platform for promoting tumor treatment. Int. J. Nanomed..

[187] Li R., He Y., Zhang S., Qin J., Wang J. (2018). Cell membrane-based nanoparticles: A new biomimetic platform for tumor diagnosis and treatment. Acta Pharm. Sin. B.

[188] Fang R.H., Kroll A.V., Gao W., Zhang L. (2018). Cell membrane coating nanotechnology. Adv. Mater..

[189] Xia Q., Zhang Y., Li Z., Hou X., Feng N. (2019). Red blood cell membrane-camouflaged nanoparticles: A novel drug delivery system for antitumor application. Acta Pharm. Sin. B.

[190] Velasco-Aguirre C., Morales F., Gallardo-Toledo E., Guerrero S., Giralt E., Araya E., Kogan M.J. (2015). Peptides and proteins used to enhance gold nanoparticle delivery to the brain: Preclinical approaches. Int. J. Nanomed..

[191] Su J., Sun H., Meng Q., Yin Q., Tang S., Zhang P., Chen Y., Zhang Z., Yu H., Li Y. (2016). Long Circulation red-blood-cell-mimetic nanoparticles with peptide-enhanced tumor penetration for simultaneously inhibiting growth and lung metastasis of breast cancer. Adv. Funct. Mater..

[192] Rao L., Bu L.L., Xu J.H., Cai B., Yu G.T., Yu X., He Z., Huang Q., Li A., Guo S.S. (2015). Red blood cell membrane as a biomimetic nanocoating for prolonged circulation time and reduced accelerated blood clearance. Small.

[193] Hu C.M., Fang R.H., Wang K.C., Luk B.T., Thamphiwatana S., Dehaini D., Nguyen P., Angsantikul P., Wen C.H., Kroll A.V. (2015). Nanoparticle biointerfacing by platelet membrane cloaking. Nature.

[194] Xiong K., Wei W., Jin Y., Wang S., Zhao D., Wang S., Gao X., Qiao C., Yue H., Ma G. (2016). Biomimetic immuno-magnetosomes for high-performance enrichment of circulating tumor cells. Adv. Mater..

[195] Xuan M., Shao J., Dai L., He Q., Li J. (2015). Macrophage cell membrane camouflaged mesoporous silica nanocapsules for in vivo cancer therapy. Adv. Healthc. Mater..

[196] Molinaro R., Corbo C., Martinez J.O., Taraballi F., Evangelopoulos M., Minardi S., Yazdi I.K., Zhao P., De Rosa E., Sherman M.B. (2016). Biomimetic proteolipid vesicles for targeting inflamed tissues. Nat. Mater..

[197] Zhu J.Y., Zheng D.W., Zhang M.K., Yu W.Y., Qiu W.X., Hu J.J., Feng J., Zhang X.Z. (2016). Preferential cancer cell self-recognition and tumor self-targeting by coating nanoparticles with homotypic cancer cell membranes. Nano Lett..

[198] Nitzsche F., Müller C., Lukomska B., Jolkkonen J., Deten A., Boltze J. (2017). Concise review: MSC adhesion cascade-insights into homing and transendothelial migration. Stem Cells.

[199] Wu H.H., Zhou Y., Tabata Y., Gao J.Q. (2019). Mesenchymal stem cell-based drug delivery strategy: From cells to biomimetic. J. Control. Release.

[200] Gao W., Fang R.H., Thamphiwatana S., Luk B.T., Li J., Angsantikul P., Zhang Q., Hu C.M., Zhang L. (2015). Modulating antibacterial immunity via bacterial membrane-coated nanoparticles. Nano Lett..

[201] Micoli F., MacLennan C.A. (2020). Outer membrane vesicle vaccines. Semin. Immunol..

[202] Pan H., Zheng M., Ma A., Liu L., Cai L. (2021). Cell/bacteria-based bioactive materials for cancer immune modulation and precision therapy. Adv. Mater..

[203] Yang J., Wang F., Lu Y., Qi J., Deng L., Sousa F., Sarmento B., Xu X., Cui W. (2019). Recent advance of erythrocyte-mimicking nanovehicles: From bench to bedside. J. Control. Release.

[204] Castro F., Martins C., Silveira M.J., Moura R.P., Pereira C.L., Sarmento B. (2021). Advances on erythrocyte-mimicking nanovehicles to overcome barriers in biological microenvironments. Adv. Drug Deliv. Rev..

[205] Hu C.M., Zhang L., Aryal S., Cheung C., Fang R.H., Zhang L. (2011). Erythrocyte membrane-camouflaged polymeric nanoparticles as a biomimetic delivery platform. Proc. Natl. Acad. Sci. USA.

[206] Fang R.H., Hu C.M., Chen K.N., Luk B.T., Carpenter C.W., Gao W., Li S., Zhang D.E., Lu W., Zhang L. (2013). Lipid-insertion enables targeting functionalization of erythrocyte membrane-cloaked nanoparticles. Nanoscale.

[207] Zou Y., Liu Y., Yang Z., Zhang D., Lu Y., Zheng M., Xue X., Geng J., Chung R., Shi B. (2018). Effective and targeted human orthotopic glioblastoma xenograft therapy via a multifunctional biomimetic nanomedicine. Adv. Mater..

[208] Lee N.H., You S., Taghizadeh A., Taghizadeh M., Kim H.S. (2022). Cell membrane-cloaked nanotherapeutics for targeted drug delivery. Int. J. Mol. Sci..

[209] Shi J., Yu W., Xu L., Yin N., Liu W., Zhang K., Liu J., Zhang Z. (2020). Bioinspired nanosponge for salvaging ischemic stroke via free radical scavenging and self-adapted oxygen regulating. Nano Lett..

[210] Liu P., Zhang T., Li C., Zhang Y., Zhou Z., Zhao Z., Chen Q., Sun T., Jiang C. (2023). Bioinspired nanoerythrocytes for metabolic microenvironment remodeling and long-term prognosis promoting of acute ischemic stroke. Nano Today.

[211] Lv W., Xu J., Wang X., Li X., Xu Q., Xin H. (2018). Bioengineered boronic ester modified dextran polymer nanoparticles as reactive oxygen species responsive nanocarrier for ischemic stroke treatment. ACS Nano.

[212] Glassman P.M., Hood E.D., Ferguson L.T., Zhao Z., Siegel D.L., Mitragotri S., Brenner J.S., Muzykantov V.R. (2021). Red blood cells: The metamorphosis of a neglected carrier into the natural mothership for artificial nanocarriers. Adv. Drug Deliv. Rev..

[213] Thon J.N., Italiano J.E. (2012). Platelets: Production, morphology and ultrastructure. Handb. Exp. Pharmacol..

[214] Lu Y., Hu Q., Jiang C., Gu Z. (2019). Platelet for drug delivery. Curr. Opin. Biotechnol..

[215] Wang S., Duan Y., Zhang Q., Komarla A., Gong H., Gao W., Zhang L. (2020). Drug targeting via platelet membrane-coated nanoparticles. Small Struct..

[216] Cui J.-W., Feng H.-C., Xu C., Jiang D.-Y., Zhang K.-H., Gao N.N., Wang Y., Tian H., Liu C. (2023). Platelet membrane-encapsulated ginkgolide B biomimetic nanoparticles for the treatment of ischemic stroke. ACS Appl. Nano Mater..

[217] Zhao C., Chen Q., Li W., Zhang J., Yang C., Chen D. (2022). Multi-functional platelet membrane-camouflaged nanoparticles reduce neuronal apoptosis and regulate microglial phenotype during ischemic injury. Appl. Mater. Today.

[218] Li M., Liu Y., Chen J., Liu T., Gu Z., Zhang J., Gu X., Teng G., Yang F., Gu N. (2018). Platelet bio-nanobubbles as microvascular recanalization nanoformulation for acute ischemic stroke lesion theranostics. Theranostics.

[219] Li M., Li J., Chen J., Liu Y., Cheng X., Yang F., Gu N. (2020). Platelet membrane biomimetic magnetic nanocarriers for targeted delivery and in situ generation of nitric oxide in early ischemic stroke. ACS Nano.

[220] Xu J., Wang X., Yin H., Cao X., Hu Q., Lv W., Xu Q., Gu Z., Xin H. (2019). Sequentially site-specific delivery of thrombolytics and neuroprotectant for enhanced treatment of ischemic stroke. ACS Nano.

[221] Xu J., Zhang Y., Xu J., Liu G., Di C., Zhao X., Li X., Li Y., Pang N., Yang C. (2020). Engineered nanoplatelets for targeted delivery of plasminogen activators to reverse thrombus in multiple mouse thrombosis models. Adv. Mater..

[222] Wang S., Wang R., Meng N., Guo H., Wu S., Wang X., Li J., Wang H., Jiang K., Xie C. (2020). Platelet membrane-functionalized nanoparticles with improved targeting ability and lower hemorrhagic risk for thrombolysis therapy. J. Control. Release.

[223] Wang C., Yang X., Jiang Y., Qi L., Zhuge D., Xu T., Guo Y., Deng M., Zhang W., Tian D. (2022). Targeted delivery of fat extract by platelet membrane-cloaked nanocarriers for the treatment of ischemic stroke. J. Nanobiotechnol..

[224] Chen L., Zhou Z., Hu C., Maitz M.F., Yang L., Luo R., Wang Y. (2022). Platelet membrane-coated nanocarriers targeting plaques to deliver anti-CD47 antibody for atherosclerotic therapy. Research.

[225] Kong J., Zou R., Chu R., Hu N., Liu J., Sun Y., Ge X., Mao M., Yu H., Wang Y. (2024). An Ultrasmall Cu/Cu_2_O nanoparticle-based diselenide-bridged nanoplatform mediating reactive oxygen species scavenging and neuronal membrane enhancement for targeted therapy of ischemic stroke. ACS Nano.

[226] Quan X., Han Y., Lu P., Ding Y., Wang Q., Li Y., Wei J., Huang Q., Wang R., Zhao Y. (2022). Annexin V-modified platelet-biomimetic nanomedicine for targeted therapy of acute ischemic stroke. Adv. Healthc. Mater..

[227] Wang D., Wang S., Zhou Z., Bai D., Zhang Q., Ai X., Gao W., Zhang L. (2022). White blood cell membrane-coated nanoparticles: Recent development and medical applications. Adv. Healthc. Mater..

[228] Chu D., Dong X., Shi X., Zhang C., Wang Z. (2018). Neutrophil-based drug delivery systems. Adv. Mater..

[229] He W., Kapate N., Shields C.W.t., Mitragotri S. (2020). Drug delivery to macrophages: A review of targeting drugs and drug carriers to macrophages for inflammatory diseases. Adv. Drug Deliv. Rev..

[230] Perez-de-Puig I., Miró-Mur F., Ferrer-Ferrer M., Gelpi E., Pedragosa J., Justicia C., Urra X., Chamorro A., Planas A.M. (2015). Neutrophil recruitment to the brain in mouse and human ischemic stroke. Acta Neuropathol..

[231] Tang C., Wang Q., Li K., Li X., Wang C., Xue L., Ju C., Zhang C. (2021). A neutrophil-mimetic magnetic nanoprobe for molecular magnetic resonance imaging of stroke-induced neuroinflammation. Biomater. Sci..

[232] Dong X., Gao J., Zhang C.Y., Hayworth C., Frank M., Wang Z. (2019). Neutrophil membrane-derived nanovesicles alleviate inflammation to protect mouse brain injury from ischemic stroke. ACS Nano.

[233] Liu S., Xu J., Liu Y., You Y., Xie L., Tong S., Chen Y., Liang K., Zhou S., Li F. (2022). Neutrophil-biomimetic “Nanobuffer” for remodeling the microenvironment in the infarct core and protecting neurons in the penumbra via neutralization of detrimental factors to treat ischemic stroke. ACS Appl. Mater. Interfaces.

[234] Feng L., Dou C., Xia Y., Li B., Zhao M., Yu P., Zheng Y., El-Toni A.M., Atta N.F., Galal A. (2021). Neutrophil-like cell-membrane-coated nanozyme therapy for ischemic brain damage and long-term neurological functional recovery. ACS Nano.

[235] Gao C., Huang Q., Liu C., Kwong C.H.T., Yue L., Wan J.B., Lee S.M.Y., Wang R. (2020). Treatment of atherosclerosis by macrophage-biomimetic nanoparticles via targeted pharmacotherapy and sequestration of proinflammatory cytokines. Nat. Commun..

[236] Wu Y., Wan S., Yang S., Hu H., Zhang C., Lai J., Zhou J., Chen W., Tang X., Luo J. (2022). Macrophage cell membrane-based nanoparticles: A new promising biomimetic platform for targeted delivery and treatment. J. Nanobiotechnol..

[237] Qu Y., Chu B., Li J., Deng H., Niu T., Qian Z. (2023). Macrophage-biomimetic nanoplatform-based therapy for inflammation-associated diseases. Small Methods.

[238] Su Y., Guo C., Chen Q., Guo H., Wang J., Kaihang M., Chen D. (2022). Novel multifunctional bionanoparticles modified with sialic acid for stroke treatment. Int. J. Biol. Macromol..

[239] Kroll A.V., Fang R.H., Jiang Y., Zhou J., Wei X., Yu C.L., Gao J., Luk B.T., Dehaini D., Gao W. (2017). Nanoparticulate delivery of cancer cell membrane elicits multiantigenic antitumor immunity. Adv. Mater..

[240] Fang R.H., Gao W., Zhang L. (2023). Targeting drugs to tumours using cell membrane-coated nanoparticles. Nat. Rev. Clin. Oncol..

[241] Rao L., Yu G.-T., Meng Q.-F., Bu L., Tian R., Lin L.-s., Deng H., Yang W., Zan M., Ding J. (2019). Cancer cell membrane-coated nanoparticles for personalized therapy in patient-derived xenograft models. Adv. Funct. Mater..

[242] Yang R., Xu J., Xu L., Sun X., Chen Q., Zhao Y., Peng R., Liu Z. (2018). Cancer cell membrane-coated adjuvant nanoparticles with mannose modification for effective anticancer vaccination. ACS Nano.

[243] Ochyl L.J., Bazzill J.D., Park C., Xu Y., Kuai R., Moon J.J. (2018). PEGylated tumor cell membrane vesicles as a new vaccine platform for cancer immunotherapy. Biomaterials.

[244] Fang R.H., Hu C.M., Luk B.T., Gao W., Copp J.A., Tai Y., O’Connor D.E., Zhang L. (2014). Cancer cell membrane-coated nanoparticles for anticancer vaccination and drug delivery. Nano Lett..

[245] Xu J., Lv J., Zhuang Q., Yang Z., Cao Z., Xu L., Pei P., Wang C., Wu H., Dong Z. (2020). A general strategy towards personalized nanovaccines based on fluoropolymers for post-surgical cancer immunotherapy. Nat. Nanotechnol..

[246] He W., Mei Q., Li J., Zhai Y., Chen Y., Wang R., Lu E., Zhang X.Y., Zhang Z., Sha X. (2021). Preferential targeting cerebral ischemic lesions with cancer cell-inspired nanovehicle for ischemic stroke treatment. Nano Lett..

[247] Thanuja M.Y., Anupama C., Ranganath S.H. (2018). Bioengineered cellular and cell membrane-derived vehicles for actively targeted drug delivery: So near and yet so far. Adv. Drug Deliv. Rev..

[248] Wu H., Jiang X., Li Y., Zhou Y., Zhang T., Zhi P., Gao J.-Q. (2020). Engineering stem cell derived biomimetic vesicles for versatility and effective targeted delivery. Adv. Funct. Mater..

[249] Yang H., Han M., Li J., Ke H., Kong Y., Wang W., Wang L., Ma W., Qiu J., Wang X. (2022). Delivery of miRNAs through metal-organic framework nanoparticles for assisting neural stem cell therapy for ischemic stroke. ACS Nano.

[250] Zhao T., Zhu T., Xie L., Li Y., Xie R., Xu F., Tang H., Zhu J. (2022). Neural stem cells therapy for ischemic stroke: Progress and challenges. Transl. Stroke Res..

[251] Sakata H., Niizuma K., Yoshioka H., Kim G.S., Jung J.E., Katsu M., Narasimhan P., Maier C.M., Nishiyama Y., Chan P.H. (2012). Minocycline-preconditioned neural stem cells enhance neuroprotection after ischemic stroke in rats. J. Neurosci..

[252] Wu H., Jiang X., Li Y., Dong Y., Zheng J., Li L., Li Y., Wang J., Lin X., Zhang X. (2023). Hybrid stem cell-derived bioresponsive vesicles for effective inflamed blood-brain barrier targeting delivery. Nano Today.

[253] Harrell C.R., Jovicic N., Djonov V., Arsenijevic N., Volarevic V. (2019). Mesenchymal stem cell-derived exosomes and other extracellular vesicles as new remedies in the therapy of inflammatory diseases. Cells.

[254] Sun Y., Jiang X., Gao J. (2024). Stem cell-based ischemic stroke therapy: Novel modifications and clinical challenges. Asian J. Pharm. Sci..

[255] Cencioni C., Capogrossi M.C., Napolitano M. (2012). The SDF-1/CXCR4 axis in stem cell preconditioning. Cardiovasc. Res..

[256] Ma J., Zhang S., Liu J., Liu F., Du F., Li M., Chen A.T., Bao Y., Suh H.W., Avery J. (2019). Targeted drug delivery to stroke via chemotactic recruitment of nanoparticles coated with membrane of engineered neural stem cells. Small.

[257] Chen G., Bai Y., Li Z., Wang F., Fan X., Zhou X. (2020). Bacterial extracellular vesicle-coated multi-antigenic nanovaccines protect against drug-resistant Staphylococcus aureus infection by modulating antigen processing and presentation pathways. Theranostics.

[258] Zhang H., Wang Y., Li M., Cao K., Qi Z., Zhu L., Zhang Z., Hou L. (2022). A self-guidance biological hybrid drug delivery system driven by anaerobes to inhibit the proliferation and metastasis of colon cancer. Asian J. Pharm. Sci..

[259] Hu K., Palmieri E., Samnuan K., Ricchetti B., Oldrini D., McKay P.F., Wu G., Thorne L., Fooks A.R., McElhinney L.M. (2022). Generalized modules for membrane antigens (GMMA), an outer membrane vesicle-based vaccine platform, for efficient viral antigen delivery. J. Extracell. Vesicles.

[260] Gao F., Xu L., Yang B., Fan F., Yang L. (2019). Kill the real with the fake: Eliminate intracellular staphylococcus aureus using nanoparticle coated with its extracellular vesicle membrane as active-targeting drug carrier. ACS Infect. Dis..

[261] Li Z., Wang Y., Liu J., Rawding P., Bu J., Hong S., Hu Q. (2021). Chemically and biologically engineered bacteria-based delivery systems for emerging diagnosis and advanced therapy. Adv. Mater..

[262] Sun R., Liu M., Lu J., Chu B., Yang Y., Song B., Wang H., He Y. (2022). Bacteria loaded with glucose polymer and photosensitive ICG silicon-nanoparticles for glioblastoma photothermal immunotherapy. Nat. Commun..

[263] Zhang L., Sun H., Zhao J., Lee J., Ee Low L., Gong L., Chen Y., Wang N., Zhu C., Lin P. (2021). Dynamic nanoassemblies for imaging and therapy of neurological disorders. Adv. Drug Deliv. Rev..

[264] Zhao N., Yan L., Zhao X., Chen X., Li A., Zheng D., Zhou X., Dai X., Xu F.J. (2019). Versatile types of organic/inorganic nanohybrids: From strategic design to biomedical applications. Chem. Rev..

[265] Huang Y., Guo X., Wu Y., Chen X., Feng L., Xie N., Shen G. (2024). Nanotechnology’s frontier in combatting infectious and inflammatory diseases: Prevention and treatment. Signal Transduct. Target. Ther..

[266] Zhan Y., Dai Y., Ding Z., Lu M., He Z., Chen Z., Liu Y., Li Z., Cheng G., Peng S. (2023). Application of stimuli-responsive nanomedicines for the treatment of ischemic stroke. Front. Bioeng. Biotechnol..

[267] Dash S., Das T., Patel P., Panda P.K., Suar M., Verma S.K. (2022). Emerging trends in the nanomedicine applications of functionalized magnetic nanoparticles as novel therapies for acute and chronic diseases. J. Nanobiotechnol..

[268] Ostruszka R., Halili A., Pluháček T., Rárová L., Jirák D., Šišková K. (2024). Advanced protein-embedded bimetallic nanocomposite optimized for in vivo fluorescence and magnetic resonance bimodal imaging. J. Colloid. Interface Sci..

